# A multicenter single-arm clinical study of Chinese children’s cancer group-acute promyelocytic leukemia-2017 (CCCG-APL-2017) protocol

**DOI:** 10.1038/s41392-025-02353-1

**Published:** 2025-08-22

**Authors:** Lixian Chang, Ju Gao, Xiaoying Lei, Yingyi He, Shuquan Zhuang, Chunhuai Li, Kaizhi Weng, Lingzhen Wang, Xia Guo, Qihui Liu, Pengfei Wang, Yong Zhuang, Mei Yan, Wei Liu, Hui Chen, Min Zhang, Shuhong Shen, Xiaofan Zhu, Xiuli Ju, Li Zhang, Zhuo Wang

**Affiliations:** 1 State Key Laboratory of Experimental Hematology, National Clinical Research Center for Blood Diseases, Haihe Laboratory of Cell Ecosystem, Institute of Hematology & Blood Diseases Hospital, Chinese Academy of Medical Sciences & Peking Union Medical College, Tianjin, China; 2 Tianjin Institutes of Health Science, Tianjin, China; 3 West China Second University Hospital, Sichuan University, Chengdu, China; 4 Children’s Hospital of Chongqing Medical University, Chongqing, China; 5 Department of Pediatric Hematology/Oncology, Guangzhou Women and Children’s Medical Center, Guangzhou Medical University, Guangzhou, China; 6 Quanzhou First Hospital Affiliated to Fujian Medical University, Quanzhou, China; 7 Department of Pediatric Hematology, Children’s Medical Center, The First Hospital of Jilin University, Changchun, China; 8 Department of Pediatric Hematology, Rheumatology and Nephrology, Zhangzhou Municipal Hospital of Fujian Province, Zhangzhou, China; 9 The affiliated hospital of Qingdao University, Qingdao, China; 10 Department of Pediatrics, Qilu Hospital of Shandong University, Jinan, China; 11 The First Affiliated Hospital of Xinjiang Medical University, Ürümqi, China; 12 Children’s Hospital Affiliated to Zhengzhou University, Zhengzhou, China; 13 Tianjin Children’s Hospital, Tianjin, China; 14 Northwest women’s and children’s Hospital, Xi’an, China; 15 Department of Hematology/Oncology, Shanghai Children’s Medical Center, School of Medicine, Shanghai Jiao Tong University, National Health Committee Key Laboratory of Pediatric Hematology & Oncology, Shanghai, China

**Keywords:** Haematological cancer, Haematological cancer

## Abstract

The Realgar-Indigo Naturalis formula (RIF) is a proprietary Chinese medicine, which is one of the important drugs in the treatment of pediatric acute promyelocytic leukemia (APL). However, the dose of RIF in clinical application is not uniform and the long-term effectiveness and safety of combining RIF with all-trans retinoic acid (ATRA) in a larger population of pediatric APL patients remains undocumented. We conducted a multicenter single-arm clinical trial (ChiCTR-OIC-16010014) in China. Individuals newly diagnosed with APL were treated with CCCG-APL-2017 protocol which is based on RIF and ATRA in consolidation. The event-free survival (EFS) and overall survival (OS) outcomes were evaluated. We recruited 200 patients diagnosed with APL. The six-year OS rate was 100% in the low-risk (LR) group and 97.6% in the high-risk (HR) group. The six-year EFS rate was 98.3% in the LR group and 97.6% in the HR group. Plasma levels of arsenic remained stable after the administration of RIF at a dosage of 60 mg/kg/d for seven days and returned to baseline levels within fourteen days after discontinuation of RIF administration, which is consistent with a concentration of 135 mg/d/kg. Furthermore, controlling white blood cells (WBC) to maintain levels at or below 30 × 10^9^/L during induction therapy can decrease the incidence of induced differentiation syndrome (DS) or alleviate its symptoms. Our study demonstrated that the CCCG-APL-2017 protocol, which combines RIF with ATRA, is both effective and safe in treating children with APL.

## Introduction

Acute promyelocytic leukemia (APL), a subtype of acute myeloid leukemia, is characterized at the molecular level by the presence of the fusion gene of *PML* (promyelocytic leukemia) and *RARA* (retinoic acid receptor alpha), which arises from the t(15;17)(q24;q21) chromosomal translocation.^1,2^ This cytogenetic abnormality leads to the production of an oncogenic fusion protein that disrupts the critical pathways regulating myeloid cell differentiation, particularly by inhibiting the normal transcriptional activity of *RARA*-targeted genes. Consequently, there is an abnormal accumulation of immature promyelocytes within the bone marrow, which disrupts the normal hematopoietic process and leads to the clinical manifestations of the disease.^1,2^ Both genetic predispositions and certain environmental exposures, such as exposure to carcinogenic substances or ionizing radiation, have been identified as potential risk factors that may elevate the likelihood of developing APL.^1,2^ Clinically, APL is noted for its rapid onset and pronounced hemorrhagic tendencies, manifesting as skin ecchymosis, epistaxis, and visceral hemorrhage. The disease is also associated with complications such as disseminated intravascular coagulation (DIC) and thrombosis, contributing to a high early mortality rate.^1,2^ Historically, APL was among the most lethal forms of leukemia. However, the advent of treatments such as all-trans retinoic acid (ATRA) and arsenic trioxide (ATO) has transformed it into a curable malignancy.^1–6^ The rate of complete remission for APL ranges from 90 to 100%.^7^ Differentiation syndrome (DS), a prevalent complication associated with ATRA and ATO therapy, is presently understood to result from the induction of cytokine release by myeloid cells, the upregulation of cell-surface integrins, and the enhancement of myeloid cell adhesion to the vascular endothelium by ATRA and ATO. These processes contribute to increased vascular permeability and the release of inflammatory mediators and cytokines, culminating in excessive inflammation and consequent tissue damage.^8,9^

The Realgar-Indigo Naturalis Formula (RIF), alternatively designated as the “compound Huangdai tablet”, represents a clinically validated Chinese patent medicine whose therapeutic composition integrates four principal botanical constituents (Ore Realgar, Indigo Naturalis, Salvia Miltiorrhiza, and Radix Pseudostellariae).^10^ Developed in the 1980s, this pharmacologic formulation strategically employs Ore Realgar as its primary active agent, with the adjunctive components—Indigo Naturalis, Salvia Miltiorrhiza, and Radix Pseudostellariae—functioning to synergistically potentiate realgar’s therapeutic index.^10^ Contemporary clinical investigations have substantiated that RIF, when administered concomitantly with ATRA or conventional chemotherapeutic agents, achieves non-inferior efficacy outcomes compared to ATO protocols.^11–13^ Beyond comparable antileukemic activity, RIF-based regimens demonstrate distinct pharmacoeconomic advantages, including demonstrably reduced treatment-related expenditures and significantly abbreviated hospitalization durations relative to ATO-containing therapies.^14^ Furthermore, RIF has been demonstrated to have a considerably lesser impact on liver function than ATO.^15^ Despite these well-documented benefits, the existing body of research remains disproportionately focused on adult patients with acute promyelocytic leukemia, creating a significant evidence gap regarding its application in pediatric populations. Additionally, there is considerable variability across different treatment protocols regarding the optimal oral dosing parameters and the required duration of maintenance therapy for RIF, which highlights the need for further standardized clinical studies.

In the consolidation therapy phase of the CCLG (Chinese children leukemia group)-APL-2016 protocol, the oral dose of RIF is specified as 60 mg/kg/d, and it is administered for a duration of 14 days.^16^ In the clinical protocol described by Luo and colleagues, the oral dose of RIF was increased to 135 mg/kg per day, and the duration of oral administration was extended to 15 days during the consolidation phase.^13^ In SCCLG (south China children leukemia group)-APL protocol, RIF was administered orally at a dose of 135 mg/kg per day, achieving a concentration of 0.61 μmol/L for arsenic on day 14.^17^ To determine a more suitable dosage and application time of RIF in pediatric patients with APL, we previously conducted a study evaluating the pharmacokinetics and safety of oral RIF in pediatric APL patients. The findings indicated that a dosage of 60 mg/kg per day resulted in a higher steady-state trough concentration in children than that achieved in adults.^18^ However, the question of how much RIF should be used and for how long in pediatric patients still requires further exploration. Given that the induction of differentiation syndrome is a primary adverse reaction to initial therapy in patients with APL, it is crucial to address the occurrence and management of this syndrome. This represents a significant clinical concern that demands careful consideration and attention from healthcare providers.

In the present study, we conducted a single-arm clinical study (ChiCTR-OIC-16010014) across multiple centers in China, based on the CCCG-APL-2017 protocol. This study was designed to optimize oral chemotherapy protocols and to comprehensively analyze efficacy, long-term survival rate, and adverse reactions associated with the use of ATRA and RIF in the treatment of pediatric APL. A total of 200 pediatric patients diagnosed with APL were enrolled from ten hospitals in China. The study revealed that arsenic plasma levels stabilized when RIF was administered at a dosage of 60 mg/kg per day, and maintaining white blood cell counts at or below 30 × 10⁹/L during induction therapy significantly reduced the risk of differentiation syndrome, thereby proving the protocol’s efficacy and safety.

## Results

### Arsenic concentration

At the commencement of the CCCG-APL-2017 study, we aimed to establish real-world arsenic concentration levels by testing six pediatric patients (Supplementary Text 1). Due to the inconvenience associated with blood collection for oral RIF therapy, which is primarily administered at home, only six patients were included in the testing. Before medication, venous blood samples are taken to measure arsenic concentration. Subsequent measurements are taken at 7, 14, 21, and 28 days after commencing medication, as well as at 1, 3, 7, and 14 days after discontinuation. Our findings indicate that arsenic concentration increases with prolonged oral administration exceeding 60 mg/kg/day; however, it stabilizes after seven days. Furthermore, arsenic concentration returns close to pre-medication levels after a withdrawal period of fourteen days. This suggests that the toxic component of RIF-arsenic is metabolized naturally post-withdrawal and poses no further risk (Supplementary Fig. 1). The specific concentrations of arsenic can be found in Supplementary Table 1. The mean arsenic concentrations measured at 7, 14, 21, and 28 days following the administration of RIF were 55.4 ng/ml, 63.8 ng/ml, 72.7 ng/ml, and 74 ng/ml, respectively. The proportions of samples exhibiting arsenic concentrations that reached or exceeded 45.7 ng/ml (0.61 μmol/L) at each time point were recorded as follows: 66.7%, 83.3%, 83.3%, and 80%.

### Patient characteristics

**Fig. 1:**
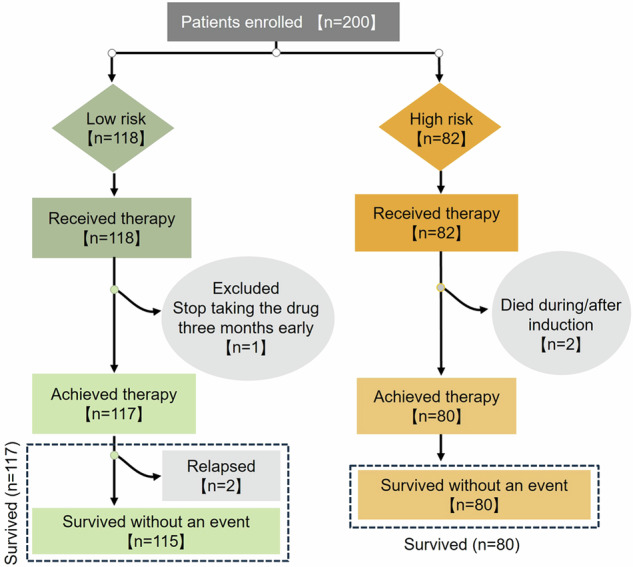
Flow chart for inclusion and treatment outcomes of APL patients. There are 200 APL patients from 10 hospitals enrolled, with 82 in the high-risk (HR) group and 118 in the low-risk (LR) group. One child in the LR group was withdrawn due to stopping taking the drug 3 months earlier. Ultimately, a total of 199 patients completed the treatment and follow-up. Two children in the HR group died, and two children in the LR group relapsed

There are a total of 200 children from 10 hospitals were enrolled, with 82 in the high-risk (HR) group (47 males and 35 females) and 118 in the low-risk (LR) group (69 males and 49 females). All Parents agreed to enroll their children in the CCCG-APL-2017 protocol and signed the informed consent form, one child in the LR group was withdrawn due to stopped taking the drug 3 months earlier. Ultimately, a total of 199 patients completed the treatment and follow-up (Fig. 1). The *PML/RARA* fusion gene status and chromosomal karyotype data are detailed in Supplementary Table 2. The baseline characteristics of these patients are presented in Table 1. No notable differences were observed between the two groups concerning gender, age, initial clinical symptoms, or family history. Nevertheless, a significant disparity was found in the newly diagnosed level of WBC and proportion of naive cells in peripheral blood between the groups (Table 1). Aside from this variation, baseline information was consistent between both groups.

**Table 1:** Basic information on enrolled patients

Characteristics	LR	HR	*p* value
***n***	117	82	
**Gender, n (%)**			0.91
Female	49 (41.9%)	35 (42.7%)	
Male	68 (58.1%)	47 (57.3%)	
**Age (year), median (IQR)**	9 (7, 11)	10 (6.25, 12)	0.38
**WBC (×10**^**9**^**L), median (IQR)**	2.8 (1.83, 4.54)	23.17 (13.57, 54.29)	<0.001
**HB (g/L), mean** **±** **SD**	81.97 ± 20.86	76.51 ± 22.99	0.08
**PLT (×10**^**9**^**L), median (IQR)**	33 (18, 58)	37 (16, 58.5)	0.94
**Proportion of naive cells in peripheral blood (%), median (IQR)**	15 (3, 42)	69.5 (40, 84)	<0.001
**Fever, n (%)**			0.09
yes	77 (65.8%)	63 (76.8%)	
no	40 (34.2%)	19 (23.2%)	
**Hemorrhage, n (%)**			0.13
no	37 (31.6%)	18 (22.0%)	
yes	80 (68.4%)	64 (78.0%)	
**Arthralgia, n (%)**			0.60
no	106 (90.6%)	76 (92.7%)	
yes	11 (9.4%)	6 (7.3%)	
**Central nervous system leukemia, n (%)**			0.47
yes	1 (0.9%)	1 (1.2%)	
no	116 (99.1%)	80 (97.6%)	
UN	0 (0.0%)	1 (1.2%)	
**Family history of tumors or Blood disorders, n (%)**			0.99
no	113 (96.6%)	80 (97.6%)	
yes	4 (3.4%)	2 (2.4%)	
**Karyotype, n (%)**			0.70
no	5 (4.3%)	2 (2.4%)	
yes	112 (95.7%)	80 (97.6%)	

*HR* high-risk, *LR* low-risk, *WBC* white blood cell, *HB* hemoglobin, *PLT* platelet, *IQR* interquartile range (Q1, Q3), *SD* standard deviation, *UN* uncertainty

### Efficacy

**Fig. 2:**
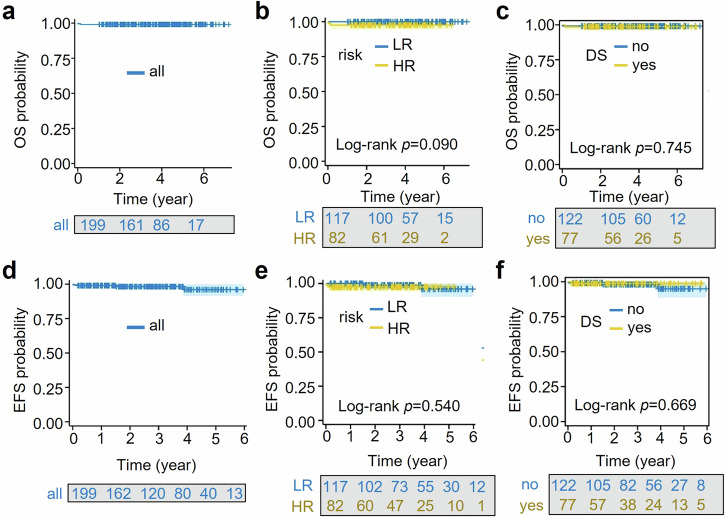
Survival prognosis analysis of risk and DS. **a** We conducted a prognosis analysis of OS for all patients. **b**, **c** OS prognosis analyses were performed for patients in different groups of risk (HR/LR) or DS (yes/no). **d**–**f** Prognosis analyses for EFS were conducted as well. The Kaplan-Meier survival curves with *p* value were provided. The number of patients at risk at each time is displayed below the respective survival curves. HR high-risk, LR low-risk

The 6-year OS rate for total APL patients (Fig. 2a) was 99.5%, while the HR group had a rate of 97.6% (Fig. 2b). There were 2 dead cases, one of which died at first diagnosis due to cerebral hemorrhage during induction therapy. Another patient died due to septicemia during the first phase of consolidation therapy. The 6-year OS for the LR group was 100%, with no statistical difference observed between the two groups (Fig. 2b). DS did not have an impact on OS in either LR or HR patients (Fig. 2c). The 6-year total EFS was 98% for APL patients (Fig. 2d), with a rate of EFS at 97.6% for HR patients and two associated deaths (Fig. 2e). The LR group achieved an EFS of 98.3%, with two cases of relapse, showing no significant difference compared to the HR group’s performance (Fig. 2e). One relapsed patient had a *PML/RARA* fusion gene level of 46.06% at diagnosis, which dropped to 0 after induction therapy. However, molecular recurrence occurred 2 years after chemotherapy was discontinued. Another patient had a positive *PML/RARA* fusion gene at diagnosis, with no quantification data. This patient had no detectable minimal residual disease (MRD) after induction therapy and consolidation 1, and MRD was negative with a quantification of 0 after consolidation 2. However, bone marrow relapsed 10 months after chemotherapy was stopped. Both patients tested negatively for FLT3-ITD (FMS-like tyrosine kinase 3—internal tandem duplication) mutation, and unfortunately, neither underwent next-generation sequencing. Patients experiencing relapses were treated with the HR group program of CCCG-APL-2017 and achieved prolonged survival. DS did not affect EFS (Fig. 2f).

Additionally, we analyzed different genders as well as whether induced differentiation syndrome occurred in different genders regarding its impact on OS and EFS. Our findings indicated that gender-specific occurrence of induced differentiation syndrome has no impact on OS and EFS outcomes (Supplementary Fig. 2).

### PML/RARA gene

**Fig. 3:**
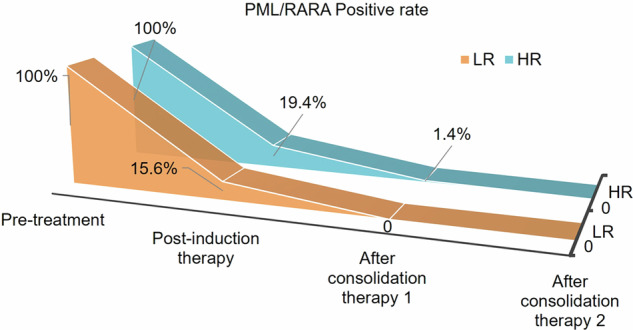
Positive rate of *PML/RARA* fusion gene during therapy. We analyzed the positive rate of *PML/RARA* fusion gene in patients at different stages before and after treatment and showed it through stereogram. HR high-risk, LR low-risk, PML/RARA promyelocytic leukemia/retinoic acid receptor alpha

Following induction therapy, 109 patients in the LR group and 72 patients in the HR group underwent testing for the *PML/RARA* fusion gene. The positive results were found in 31 patients, comprising 15.6% (17/109) in the LR group and 19.4% (14/72) in the HR group. After the first consolidation therapy, all patients in the LR group achieved *PML/RARA* fusion gene negativity, while one patient in the HR group failed to convert the gene. This patient subsequently achieved gene negativity after undergoing a second consolidation treatment (Fig. 3).

### Differentiation syndrome

We analyzed the incidence, clinical manifestations, and risk factors of differentiation syndrome (DS) in induction therapy. During induction therapy, the overall incidence of DS was 38.7% (77/199). Specifically, the incidence of DS was 45.1% (37/82) among HR cases and 34.2% (40/117) among LR cases. All cases of DS were indeterminate,^9^ and no statistically significant difference was observed in the incidence between the two groups (Supplementary Table 3). No deaths related to DS were reported. Upon analyzing the risk factors influencing DS, it was found that the factors of WBC and PLT (platelet) were associated with DS occurrence (Supplementary Table 4).

**Fig. 4:**
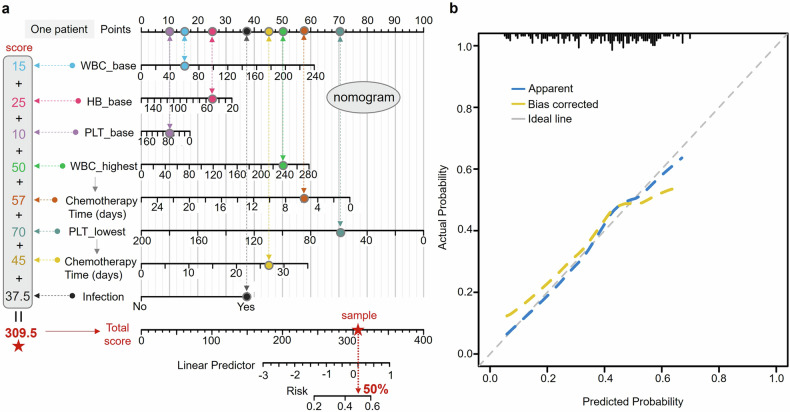
Nomogram and calibration curve of differentiation syndrome. **a** The nomogram integrates key clinical variables to estimate the probability of differentiation syndrome. Each variable is assigned a point value on the upper scale (e.g., 15 for WBC_base and 25 for HB_base). The total score, derived from summing up the points for all variables (e.g., a cumulative score of 309.5), is then referenced on the lower axis to determine the corresponding predicted probability (e.g., approximately 50% risk in this instance). **b** The calibration curve evaluates the agreement between predicted and observed probabilities. The apparent line (blue dashed) represents uncorrected predictions, the bias corrected line (yellow dashed) shows adjusted predictions, and the ideal line (gray dashed) indicates perfect calibration. WBC white blood cell, HB hemoglobin, PLT platelet

In addition, the nomogram tool enables clinicians to evaluate individual patient risk for specific diseases, thereby facilitating informed clinical decision-making and patient management.^19–21^ Thus, we also incorporated key clinical variables—namely WBC_base, HB_base, PLT_base, duration of chemotherapy, and infection status—into the development of a nomogram designed to predict the risk of differentiation syndrome (Fig. 4). For instance, each variable of one patient is assigned a specific score based on its value, and these scores are aggregated to yield a total score of 309.5. This total score is then translated into a risk probability of approximately 50%, as depicted at the bottom of the nomogram (Fig. 4a). Furthermore, we generated a calibration curve to assess the concordance between the nomogram’s predicted probabilities of differentiation syndrome and the actual observed probabilities. As illustrated in Fig. 4b, the predicted probabilities closely match the observed probabilities, demonstrating good calibration and reliability of the model, which enhances the nomogram’s clinical applicability.

### Treatment toxicities

We assessed the toxic side effects according to CTCAE (common terminology criteria for adverse events) 4.0 during induction treatment phase (Supplementary Table 5). 89.9% of patients (179/199) experienced grade 3–4 anemia, while 90.95% experienced grade 3–4 neutropenia (181/199), and 86.9% experienced grade 3–4 thrombocytopenia (173/199). Additionally, the incidence of disseminated DIC is 37.2% (74/199) with grade 3–5 DIC occurring in 15.1% (30/199) of patients, with one patient in the HR group succumbing to grade 5 DIC with intracranial hemorrhage. The overall incidence of bleeding was 36.2% (72/199), with severe bleeding (grade 3–5) occurring in 5% (10/199). The bleeding rates were significantly higher in the HR group at 51.2% (42/82), compared to 25.6% (30/117) in the LR group. Notably, abnormal coagulation remains a significant factor contributing to early death. The incidence of thrombus was 2% (4/199). In the LR group, the incidence of thrombus was 1.7% (2/117), with one case of grade 1 accompanied by grade 4 hemorrhage and DIC, and one case of grade 2 without hemorrhage and DIC. In the HR group, the incidence of thrombus was 2.4% (2/82), all of which were grade 2 and accompanied by grade 2 hemorrhage, with one case of grade 1 DIC and one case of grade 3 DIC. Regarding cardiotoxicity, only 6% (12/199) of patients experienced prolonged corrected QT intervals at grades 1–2. The cardiotoxicity incidence was 7.3% (6/82) in the HR group, and 5.1% (6/117) in the LR group, respectively. No notable distinction was observed between the two groups. All these 12 patients had grade 1–2 cardiac toxicity and could recover with supportive treatment. Additionally, 7% (14/199) had elevated serum ALT/AST (alanine transaminase / aspartate aminotransferase) levels at a grade 3 level, while hyperbilirubinemia, was found in grades 1–2 for about 6.5% (13/199) of patients.

Due to the distinct treatment protocols for the LR and HR groups during the consolidation and maintenance phases, we performed a separate analysis of the toxic reactions in each group. In the LR group, the incidence of hematological toxicity and hepatotoxicity was significantly elevated during the initial stage of consolidation treatment compared to other treatment stages (Supplementary Fig. 3 left panel and Supplementary Table 6*, p* < 0.05). Additionally, in the second stage of consolidation treatment, QTc (corrected QT interval) prolongation was observed in only one patient (grade 1) of LR group (Supplementary Table 6). In contrast, the HR group exhibited a significant increase in the incidences of hematological toxicity, cardiotoxicity, hepatotoxicity, nausea, vomiting, and infectious fever during the consolidation treatment stage (Supplementary Fig. 3 right panel and Supplementary Table 7, *p* < 0.05), which may be attributed to the infusion of Idarubicin. Notably, ALT and AST levels remained persistently elevated, with this abnormality continuing until the second stage of maintenance treatment (Supplementary Fig. 3 right panel). During the first and second stages of consolidation treatment, five patients (grade 1) of HR group displayed prolonged QTc (Supplementary Table 7). More critically, there was one fatality due to sepsis during the first stage of consolidation treatment. A comprehensive analysis indicates that the overall toxicity level of patients in the HR group was higher than that in the LR group throughout the entire consolidation and maintenance treatment period.

### Infection

In our study, infections were classified into two distinct categories. The first category encompassed fever associated with a clear focus on infection, such as oral, pulmonary, or bloodstream infections, irrespective of the presence of DS. This type of fever was substantiated by clinical or laboratory evidence indicating an infection. The second category included fever without a discernible focus of infection and with DS excluded; this typically occurred during granulocytopenia and is referred to as isolated fever (i.e., “no obvious infection” in Supplementary Fig. 4). In clinical practice, we generally consider that fever induced solely by ATRA and arsenic agents during the early treatment phase—regardless of accompanying muscle pain and in the absence of an evident infectious focus—may signify induction syndrome and can be effectively managed with corticosteroids. In contrast, fever accompanied by a clear infectious source or positive indicators related to infection (e.g., blood culture positivity) is categorized as infectious. The overall infection rate was 76% (151/199) during induction therapy, and there was no notable distinction between the LR and HR categories in terms of infection prevalence (Supplementary Table 8).

Among the sites of infection, pulmonary infections were the most common, with HR patients being more susceptible to developing pulmonary infections (Supplementary Fig. 4). Pathogens were detected in 23 patients, including four cases of mycoplasma pneumoniae, of which two had other infections, one had aureus septicemia, and one had herpes simplex virus. Three cases of pulmonary fungal infections, and six cases of pulmonary bacterial infections, which included Streptococcus, Haemophilus influenzae, MRSA (Methicillin-resistant Staphylococcus aureus) + perianal swab enzyme-producing Escherichia coli from a throat swab, Stenotrophomonas maltophilia, Streptococcus pneumoniae. Additionally, there were four cases of viral infections: herpes simplex virus (2 cases), varicella-zoster virus (1 case), and unknown virus (1 case). Five cases of sepsis caused by Micrococcus luteus, Streptococcus agalactiae subsp. agalactiae, Staphylococcus aureus, Staphylococcus hominis subsp. hominis, and MRSA. Furthermore, there was one case involving intestinal trichomonas.

### Gene mutation

Out of 199 patients, mutation data for specific genes was obtained from 138 individuals. Among these, 34 patients exhibited no gene mutations, while mutations such as FLT3-ITD, FLT3-TKD (FMS-like tyrosine kinase 3—tyrosine kinase domain), NRAS (Neuroblastoma RAS viral oncogene homolog), KRAS (Kirsten rat sarcoma viral oncogene homolog), and WT1 (Wilms tumor 1) were detected in 104 patients. Supplementary Fig. 5 presents a heat map of these mutant genes, showing that the mutation frequencies of FLT3-ITD and FLT3-TKD were higher in the HR group than in the LR group. Correlation analysis (Supplementary Table 9) revealed that the HR group had a higher FLT3-ITD mutation rate (36.5% vs. 13.3%, *p* = 0.001) and a higher FLT3-TKD mutation rate (30.2% vs. 10.7%, *p* = 0.004) compared to the LR group. Additionally, no significant correlation was found between these gene mutations and the occurrence of DS (Supplementary Table 10). Notably, none of the patients with FLT3-ITD or FLT3-TKD mutations experienced a relapse; however, it is regrettable that the two patients who did relapse did not undergo next-generation sequencing.

Subsequently, we investigated whether single-gene mutations (e.g., FLT3-ITD, FLT3-TKD, NRAS) or dual-gene mutations (e.g., FLT3-ITD/TKD, FLT3-ITD/NRAS) influence the increase in WBC (WBC_highest / base) and the decrease in PLT (PLT_lowest / base) during induction therapy. The results, shown in Supplementary Fig. 6-7, indicated no significant effects.

Finally, we conducted a Gene Ontology (GO) and Kyoto Encyclopedia of Genes and Genomes (KEGG) pathway enrichment analysis using these identified mutant genes (Supplementary Fig. 8). Our analysis revealed that these gene mutations are predominantly associated with blood cell differentiation. Furthermore, the analysis indicated enrichment in categories such as DNA-binding transcription factor binding, transcription coregulator activity, and ATPase complex. A series of mutant genes linked to gene transcription, particularly those associated with the transcription regulator complex, suggests that the regulatory networks of genes involved in blood cell differentiation may play a significant role in the underlying mechanisms of APL in children.

## Discussion

Previous reports indicate that As_2_O_3_ primarily induces apoptosis at higher concentrations (0.5–2 μmol/L) and partial differentiation at lower concentrations (0.1 to 0.5 μmol/L) in APL cells.^22^ Comparing RIF at 135 mg/kg/day to ATO at 0.16 mg/kg/day, both have similar plasma arsenic levels, with troughs around 0.5–1 µmol/L.^17^ However, at 60 mg/kg/day of RIF versus 0.16 mg/kg/day of ATO, the trough levels were 11.5 µg/L for RIF and 21.7 µg/L for ATO.^12^ Our findings show that a 60 mg/kg/d oral RIF dosage results in an average arsenic concentration of 0.74 μmol/L by day 7 in children. Chen et al. reported an increase in myelocyte-like cells in bone marrow and peripheral blood examinations after 2 to 3 weeks of continuous As_2_O_3_ treatment in vivo, coinciding with a decrease in leukemic promyelocytes.^22^ To ensure efficacy, we opted for a 60 mg/kg/d oral maintenance dosage over 3–4 weeks. A higher dosage of the AS_4_S_4_ may be harmful to both liver and kidney functions, particularly in children.^18^ After drug withdrawal, arsenic levels returned to pre-treatment levels within 14 days. Therefore, our treatment protocol is to include a rest period of 2–3 weeks between different courses of treatment. This protocol supports the scientific use and dosage of RIF. After a median follow-up of 3.9 years, treatment effects remained stable. RIF can be taken orally at home, offering a more convenient and cost-effective option than ATO, reducing financial strain on families and allowing children to return to normal activities sooner.

In pediatric APL treatment, oral RIF combined with ATRA is as effective as intravenous ATO/ATRA.^11,13,16,23^ Zheng et al. reported a 99% 2-year overall survival with oral therapy per the CCLG-APL 2016 Protocol.^16^ Luo et al. found a 100% disease-free survival rate in both treatment groups after two years.^13^ Huang et al. noted a 97.6% 5-year EFS rate for both ATO and oral therapy groups in the SCCLG-APL study.^11^ Our study, with 199 patients, showed a 100% 6-year OS rate in the LR group and 97.6% in the HR group, with EFS rates of 98.3% and 97.6%, respectively. Among 199 patients, EFS was slightly higher in those with DS than without, but not significantly (Fig. 2f, *p* = 0.669). Of these patients, 130 received cytarabine, averaging 4 days of treatment (median 8 days, range 1–27) and a mean dose of 803 mg (median 700 mg, range 35–2940 mg). Cytarabine was administered to 81.8% of DS patients and 54.9% of non-DS patients (*p* < 0.001) (Supplementary Table 11). In addition, we analyzed the link between cytarabine treatment and OS/EFS. Supplementary Fig. 9 shows that patients treated with cytarabine had a more favorable trend in OS and EFS, though not statistically significant. This aligns with EFS outcomes in DS-related cases, suggesting cytarabine may improve EFS in DS patients. In induction therapy, our neutropenia rate (90.95%, grade 3 + 4) surpassed Zheng et al.‘s 80%,^16^ likely due to cytarabine and hydroxyurea. Infections during this phase are diverse and impactful, necessitating multiple blood cultures for pathogen identification and broad-spectrum antimicrobials if the cause is unclear. Pulmonary infections are most common, warranting early CT scans for recurrent fever. During the consolidation and maintenance phase, the toxicity of oral ARTA and RIF was less than ATO combined with ATRA and also lower than oral 135 mg/kg/d of RIF, especially the incidence of QTc prolongation.^11,23,24^ This might be linked to RIF’s traditional Chinese medicine properties, though further research on its compound effects is needed.

This study is designed as a single-arm investigation. Given the rarity of pediatric APL and the well-documented efficacy of combining arsenic with ATRA in treating pediatric APL patients, this approach is justified. Due to the lack of consensus regarding the combination of oral RIF with ATRA, the single-arm design was implemented to standardize the treatment of APL using RIF and ATRA, thereby facilitating increased patient enrollment. Consequently, our investigation, guided by experts from our clinical epidemiology and evidence-based medicine center, was structured as a single-arm study involving children diagnosed with APL. Furthermore, we acknowledge that the absence of comprehensive second-generation sequencing analysis and a systematic evaluation of concurrent mutations may limit the generalizability of our conclusions. Variations in clinical workflows and resource availability across participating institutions have resulted in inconsistent implementation of genomic sequencing protocols, leading to some patients not receiving sequencing. To address this limitation, we plan to conduct additional comprehensive genomic sequencing analysis, focusing on specific gene mutation sites and the mutation status of other related genes. This approach will allow for a more systematic assessment of mutational patterns and their prognostic implications, thereby enhancing the validity of our findings.

Different studies use various criteria for risk classification in APL patients. Huang et al. categorized patients into LR, intermediate-risk, and HR based on WBC and platelet levels.^11^ Zheng grouped patients into standard risk and HR based on WBC count and FLT3-ITD mutations.^16^ Since FLT3-ITD does not affect APL aggressiveness in adult,^25^ our study classified patients into LR and HR groups based solely on WBC count for simplicity and feasibility. We found FLT3-ITD and FLT3-TKD mutations are more common in the HR group (Supplementary Table 10). No patients with these mutations experienced relapse or death, suggesting they are not linked to disease recurrence but may correlate with higher WBC counts. Previous studies reported ATRA and ATO-associated DS incidence between 2.5% and 37%, with onset typically between 7 and 12 days.^8,26–29^ Severe DS appears sooner, around 6 days, while moderate cases develop around 15 days.^28^ In our study, DS incidence was 38.7%. We treated indeterminate-grade DS with glucocorticoids, hydroxyurea, and cytarabine to maintain white blood cells at 50 × 10^9^/L, effectively reducing pain and mortality. Analysis of risk factors for DS revealed that the risk increases when the WBC count rises above 30 × 10^9^/L. To mitigate the risk of DS in later stages, cytarabine should be administered when the WBC count reaches 30 × 10^9^/L, rather than waiting until it reaches 50 × 10^9^/L.

In conclusion, our findings demonstrate that the CCCG-APL-2017 protocol based on ATRA and RIF for treating pediatric APL is effective and has a low incidence of side effects. The recommended dosage of RIF is 60 mg/kg/d, with a suggested duration of 3–4 weeks. To mitigate the risk of differentiation syndrome, it is advisable to maintain WBC counts at or below 30 × 10^9^/L.

## Materials and methods

### Study design and patients

The CCCG-APL-2017 study is a multi-center, single-arm clinical trial. Its primary objective is to evaluate the EFS and OS associated with the CCCG-APL-2017 protocol, which combines RIF and ATRA for the treatment of pediatric APL. Secondary objectives include assessing the time to achieve fusion gene negativity and its corresponding rate, reducing both the incidence and severity of DS, as well as evaluating the safety profile of the RIF and ATRA combination therapy, particularly concerning the incidence and severity of adverse events. The study involved 200 children diagnosed with APL, recruited from October 2017 to June 2022. A total of 200 children from 10 hospitals were enrolled, including 55 in the State Key Laboratory of Experimental Hematology, National Clinical Research Center for Blood Diseases, 38 in Shanghai Children’s Medical Center, 34 in West China Second University Hospital, 22 in Guangzhou Women and Children’s Medical Center, 18 in Children’s Hospital of Chongqing Medical University, 15 in Qilu Hospital of Shandong University, 8 in Quanzhou First Hospital Affiliated to Fujian Medical University, 4 in the First Hospital of Jilin University. 2 in the affiliated hospital of Qingdao University and 4 in Zhangzhou Municipal Hospital of Fujian Province. Confirmation of APL diagnosis was based on bone marrow cell morphology, as well as the detection of the *PML/RARA* fusion gene or t (15;17) chromosomal translocation. The age range of participants in this research varied from 29 days to 18 years. Registration for this study was completed at the Chinese Clinical Trial Registry under the code ChiCTR-OIC-16010014. This study was approved by the Ethics Committee of Hematology Hospital, Chinese Academy of Medical Sciences (IIT2016008-EC-2). The informed consent was provided by patients’ families and/or patients themselves following the Declaration of Helsinki.

### Treatment protocol

**Fig. 5:**
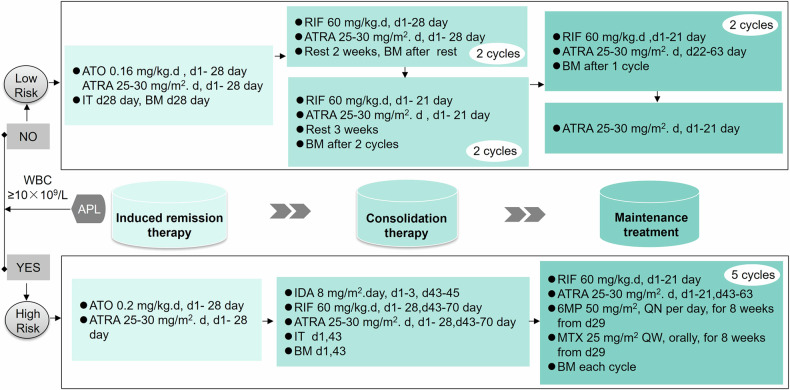
Treatment protocols of CCCG-APL-2017. The APL patients were divided into low-risk and high-risk groups according to whether the white blood cells were less than 10 × 10^9^/L before ATRA or/and ATO treatment, the patients APL patients underwent induction therapy, consolidation therapy, and maintenance therapy in three stages. APL acute promyelocytic leukemia, ATO arsenic trioxide, ATRA all-trans retinoic acid, IDA Idarubicin, IT Lumbar puncture combined with triple sheath injection, BM bone marrow aspiration, RIF Realgar-Indigo Naturalis formula, 6MP 6-Mercaptopurine, MTX methotrexate, QN quaque nocte, QW quaque week

The treatment protocols are shown in Fig. 5. Further instructions can be found in Supplementary Text 2. The risk group has been defined as follows: LR group, which includes individuals with peripheral blood WBC < 10 × 10^9^/L before ATRA or/and ATO treatment and good treatment response (*PML/RARA* negative after two consolidation treatments); HR group, which encompasses those with peripheral blood WBC ≥ 10 × 10^9^/L before ATRA or/and ATO treatment, or those with unsatisfactory treatment response (such as patients in the original LR group who do not achieve complete response after 56 days of induction therapy, or those who do not have negative *PML/RARA* after two courses of consolidation therapy, or they have positive *PML/RARA* for two consecutive times after it turns negative), or those experiencing a molecular genetic relapse. To conserve medical resources and minimize the toxic and adverse reactions of chemotherapeutic drugs, intravenous arsenic is discontinued following a response, and instead, RIF at a dosage of 60 mg/kg/d is used. For LR APL cases post-response, cytotoxic chemotherapeutic agents should be avoided while utilizing RIF (60 mg/kg/d) and ATRA (25–30 mg/m^2^). It is advised to early administer dexamethasone prophylactically to lessen the frequency and seriousness of differentiation syndrome. Patients with WBC more than 5 × 10^9^/L are treated with hydroxycarbamide 20–50 mg/kg/d for more than three days, and it is recommended to continue until WBC decreases to ≤10 × 10^9^/L. Patients with WBC ≥ 50 × 10^9^/L during ATRA + ATO therapy are given cytarabine (Ara-c) 50 mg/m^2^ intravenously for 12 h until WBC decreases to ≤ 10 × 10^9^/L.

### Sample size setting

Sample size setting was performed using the “One-Sample Logrank Tests Assuming a Weibull Model (Wu)” Procedure in PASS 2021 software. The calculation was based on 3-year EFS rates, with a historical control rate of 90% and an anticipated trial rate of 95%, α = 0.05, power = 80%, a 4-year recruitment period, and 3-year follow-up. The calculated sample size was 151 patients, adjusted to 167 considering a 10% dropout rate. Due to the COVID-19 (coronavirus disease-2019) pandemic and low incidence of pediatric APL, enrollment was delayed by 8 months. To account for potential increased dropouts during the pandemic, the dropout rate was raised to 30%, resulting in a recalculated sample size of 196, approximately 200 patients. Fortunately, no patients were lost to follow-up, likely due to the oral administration protocol allowing remote guidance during the pandemic.

### Response criteria

Complete response: <5% of bone marrow promyelocytes; absence of blasts with Auer corpuscles, the persistence of extramedullary leukemia, and no extramedullary leukemia. Absolute neutrophil count ≥1.0 × 10^9^/L, platelet count ≥100 × 10^9^/L. Partial response: 5%-25% of bone marrow promyelocytes. Molecular genetic response: Negative result for *PML/RARA* fusion gene in bone marrow as determined by nested reverse transcription-polymerase chain reaction (RT-PCR) analysis. No response: >25% bone marrow promyelocytes. Drug resistance: failure to achieve complete response after 56 days of induction therapy. Relapse: > 20% bone marrow promyelocytes after achieving a previous response. Molecular genetic relapse: RT-PCR initially shows a negative result for the *PML/RARA* fusion gene, followed by two subsequent positive results with an interval of 2–3 weeks.

### Gene mutation analysis

We systematically collected (Supplementary Table 12) and evaluated accessible genomic mutation information. By integrating patient risk strata with gene mutation profiles, we conducted a comprehensive analysis. This was followed by risk categorization and result visualization, executed using the pheatmap R package. Gene identifiers were standardized utilizing the org.Hs.eg.db R package. We performed functional enrichment analyses on GO categories, encompassing biological processes (BP), cellular components (CC), and molecular functions (MF), in conjunction with KEGG pathways. The clusterProfiler R package was employed for these analyses, with results presented through ggplot2. Furthermore, we integrated mutation data with survival outcomes and clinical metrics to investigate associations between single and dual gene mutation types.

### Statistical analysis

If the data from measurements follows a normal distribution, they should be represented as mean ± standard deviation (SD). When comparing two groups of samples, a *t* test is used. If the measurement data do not follow a normal distribution, a Mann–Whitney U test was utilized to evaluate the differences between groups. A chi-square test was performed using the stats [4.2.1] R package. For the Nomogram and standard curve analysis, we utilized the rms [6.4-0] R package to fit the model and calculate the risk scores of each variable. Additionally, we employed Resource Selection [0.3-5] to measure the calibration degree of the model. The glm function was used to construct a binary logistic model, and the rms package Calibration was utilized for analysis and visualization purposes.

We conducted survival analyses for OS and EFS. OS was defined as the time from the initiation of treatment in the single-arm trial to death from any cause. EFS was measured from the start of treatment to the first occurrence of any event, including disease progression, local or distant recurrence, or death from any cause. For the analysis, we used the Surv function to create survival objects and the survfit function to perform Kaplan-Meier analysis. Kaplan-Meier survival curves were visualized using the survminer and ggplot2 packages, and the Log-rank test was applied to assess statistical significance. A *p-*value threshold of 0.05 was set to determine statistical significance. All analyses were conducted using IBM SPSS Statistics version 20.0 and R software (version 4.2.1).

## Supplementary information

supplementary material

## Data Availability

The available raw sequence data have been deposited in the Genome Sequence Archive (GSA) at the National Genomics Data Center, China National Center for Bioinformation / Beijing Institute of Genomics, Chinese Academy of Sciences (GSA-Human: HRA012149, HRA012104) that are publicly accessible at https://ngdc.cncb.ac.cn/gsa-human. The datasets generated during this study are available upon request from the corresponding authors.
