# Supplementary material for: A multicenter single-arm clinical study of Chinese children’s cancer group-acute promyelocytic leukemia-2017 (CCCG-APL-2017) protocol
Source: Signal Transduct Target Ther. 2025 Aug 22;10:267. doi: 10.1038/s41392-025-02353-1 (PMC12370879; doi:10.1038/s41392-025-02353-1)
Supplement: Supplementary file 1 — supplementary material [file 41392_2025_2353_MOESM1_ESM.docx]

Supplementary Materials for

A multicenter single-arm clinical study of Chinese children's cancer group-acute promyelocytic leukemia-2017 (CCCG-APL-2017) protocol

Lixian Chang, Ju Gao, Xiaoying Lei, Yingyi He, Shuquan Zhuang, Chunhuai Li, Kaizhi Weng, Lingzhen Wang, Xia Guo, Qihui Liu, Pengfei Wang, Yong Zhuang, Mei Yan, Wei Liu, Hui Chen, Min Zhang, Shuhong Shen, Xiaofan Zhu, Xiuli Ju, Li Zhang, and Zhuo Wang

Correspondence to: wangzhuo111@sina.com (Zhuo Wang); zhangli1@ihcams.ac.cn (Li Zhang); jxlqlyy@163.com (Xiuli Ju)

**This PDF file includes:**

Supplementary Text 1 to 2

Figures. S1 to S9

Tables S1 to S12

Supplementary Text 1

Experimental procedure for arsenic concentration detection

Arsenic concentration test

The concentration of arsenic in collected plasma was determined using inductively coupled plasma mass spectrometry (ICP-MS). 2 mL of plasma was collected for each assay, and the specimens were stored at 4 °C and analyzed within two weeks. The total arsenic analysis was performed using an Agilent 7700 × ICP-MS equipped with a pure He octopole reaction system from Agilent Technology, USA. No polyatomic interference or argon chloride interference was observed during the analysis. For sample preparation, a 1.0 mL volume of blood was digested in 2 mL of HNO_3_ (65%) and 1 mL of H_2_O_2_ (30%) using a microwave digestion system, then diluted to a total volume of 8 mL with deionized water (Nitric acid-UP, China, BV-III grades). All sample solutions were clear, including those from blank digestions performing using the same method. The microwave digestion conditions used were as follows: 5 min at 1300 W and 160°C; 5 min at 1300 W and 200°C; 20 min at 1300 W and 200°C. The digested samples were filled to their final volumes using ultrapure water before being analyzed by ICP-MS. A standard curve covering a linear range from 0 to 20 ng/mL was generated with a detection limit of 0.01 μg/L.

Supplementary Text 2

The instruction of CCCG-APL2017 protocol

Risk grouping, further instructions and treatment response criteria

The risk group has been defined as follows: Low-risk group, which includes individuals with peripheral blood WBC < 10×10^9^/L before ATRA or/and ATO treatment and good treatment response (*PML/RARA* negative after two consolidation treatments); High-risk group, which encompasses those with peripheral blood WBC ≥ 10×10^9^/L before ATRA or/and ATO treatment, or those with unsatisfactory treatment response (such as patients in the original low-risk group who do not achieve complete response after 56 days of induction therapy, or those who do not have negative *PML/RARA* after two courses of consolidation therapy, or they have positive *PML/RARA* for two consecutive times after it turns negative), or those experiencing a molecular genetic relapse. To conserve medical resources and minimize the toxic and adverse reactions of chemotherapeutic drugs, intravenous arsenic is discontinued following a response, and instead, RIF at a dosage of 60 mg/kg/d is used. For low-risk APL cases post-response, cytotoxic chemotherapeutic agents should be avoided while utilizing RIF (60 mg/kg/d) and ATRA (25-30 mg/m^2^). It is advised to early administer dexamethasone prophylactically to lessen the frequency and seriousness of differentiation syndrome.

Treatment response criteria

Complete response: < 5% of bone marrow promyelocytes; absence of blasts with Auer corpuscles, the persistence of extramedullary leukemia, and no extramedullary leukemia. Absolute neutrophil count ≥ 1.0 × 10^9^/L, platelet count ≥ 100×10^9^/L. Partial response: 5%-25% of bone marrow promyelocytes. Molecular genetic response: Negative result for *PML*/*RARA* fusion gene as determined by RT-PCR analysis. No response: > 25% bone marrow promyelocytes. Drug resistance: failure to achieve complete response after 56 days of induction therapy. Relapses: > 20% bone marrow promyelocytes after achieving a previous response. Molecular genetic relapses: RT-PCR initially shows a negative result for the *PML*/*RARA* fusion gene, followed by two subsequent positive results with an interval of 2-3 weeks.

Low-risk group

Once APL is suspected, differentiation-inducing therapy with ATRA should be initiated as soon as possible without waiting for cytogenetic and molecular genetic findings.

1. Induced remission therapy

1. ATRA 25-30 mg/m^2^. d, orally, 2-3 times. At least 28 days until a complete bone marrow morphologic response is obtained, and up to 56 days.
2. ATO 0.16 mg/kg/d, iv drip, qd, for at least 28 days until complete morphologic remission of bone marrow is obtained, and up to 56 days.
3. Dexamethasone 2.5 mg/m^2^, bid. Used for 15 consecutive days to prevent induction syndrome.
4. Patients with WBC more than 5 × 10^9^/L are treated with hydroxycarbamide 20-50 mg/kg/d for more than 3 days, and it is recommended to continue until WBC decreases to ≤ 10 × 10^9^/L.
5. Patients with WBC ≥ 50 × 10^9^/L during ATRA + ATO therapy are given Ara-C 50 mg^/^m 2 intravenously q12h until WBC decreases to ≤ 10 × 10^9^/L.
6. The intrathecal injection should only be performed in patients with obvious CNSL manifestations whose intracranial hemorrhage can be excluded, and the intrathecal injection should be performed after the coagulation function is stable. If no immature cells are found in the cerebrospinal fluid, no further intrathecal injections will be given thereafter. If the cerebrospinal fluid examination reveals immature cells, an intrathecal injection should be given once every 3 days until the cerebrospinal fluid immature cells have disappeared.
7. On day 28 of the induced remission therapy, bone marrow puncture is performed to complete the morphological examination of bone marrow cells, and *PML/RARA* fusion gene is detected by FISH and RT-PCR to assess the cytogenetic and molecular biological response. If bone marrow morphologic complete response is not achieved, ATR+ATO therapy should be continued, and bone biopsy should be repeated every 2 weeks. In the low-risk group, after complete response is achieved at week 6 and within 6 weeks, treatment is withheld for 2 weeks, and then consolidation therapy is started. If patients still fail to achieve a response after 8 weeks, they should be transferred to the high-risk group.

2. Consolidation therapy (bone puncture prior to initiation)

1. ATRA 25-30 mg/m^2^/d, administered orally in 2-3 divided doses.
2. Compound Realgar Natural Indigo Tablet 60 mg/kg/d, po, tid.

For the first and second consolidation treatments, each course of treatment lasts for 4 weeks, with a rest of 2 weeks. For the third and fourth consolidation treatments, each course of treatment lasts for 3 weeks, with a rest of 3 weeks.

1. Triple intrathecal injection is given once on the first day. If no immature cells are found in the cerebrospinal fluid, no further intrathecal injections will be given thereafter. If the cerebrospinal fluid examination reveals immature cells, an intrathecal injection should be given once every 3 days until the cerebrospinal fluid immature cells have disappeared. Intrathecal injection is given again on the first day of the second consolidation treatment.

**Composition and dose of triple intrathecal injection**

| **Age** | **MTX** | **Ara-C** | **DX** | **NS** |
| --- | --- | --- | --- | --- |
| < 12 months | 6 mg | 15 mg | 2.5 mg | 6 mL |
| 12-36 months | 9 mg | 25 mg | 2.5 mg | 6 mL |
| ≥ 36 months | 12.5 mg (max) | 35 mg | 5.0 mg | 10 mL |

3. Maintenance treatment

Compound Realgar Natural Indigo Tablet for 3 weeks ATRA for 6 weeks Compound Realgar Natural Indigo Tablet for 3 weeks ATRA for 6 weeks Compound Realgar Natural Indigo Tablet for 3 weeks. (same dose as above)

High-risk group

Once APL is suspected, ATRA should be administered as an emergency treatment without waiting for cytogenetic and molecular genetic confirmation. If WBC > 50×10^9^/L and the diagnosis has been confirmed, ATO can be used alone and ATRA can be used jointly after the condition is stable.

Induced remission

1. ATRA 25-30 mg/m^2^.d, orally in 2-3 divided doses for at least 28 days until complete morphologic response of the bone marrow is obtained, and up to 56 days.
2. ATO 0.2 mg/kg/d, intravenous drip, at least 28 days until bone marrow morphologic response is obtained, and up to 56 days.
3. Dexamethasone 2.5 mg/m^2^ BID for 15 consecutive days to prevent induction syndrome.
4. Hydroxycarbamide 20-50 mg/kg/d is used for more than 3 consecutive days, and is recommended until WBC decreases to ≤ 10 × 10^9^/L.
5. One week later, if WBC is still ≥ 20 × 10^9^/L, and the coagulation function is stable, Ara-C 50 mg/m^2^ should be administered for at least 2 hours until WBC decreases to ≤ 10 × 10^9^/L. If WBC is 50 × 10^9/^L and the coagulation function is stable, Ara-C can be administered till the third day.
6. The assessment of bone puncture and intrathecal injection in induced remission therapy are the same as those in the low-risk group.

Consolidation therapy: (bone puncture prior to initiation)

1. ATRA 25-30 mg/m^2^. day, orally in 2-3 times, d1-28, d43-70.
2. Compound Realgar Natural Indigo Tablet 60 mg/kg/d, po, three times, d1-28, d43-70.
3. IDA 8 mg/m^2^.day, QD, d1-3, d43-45.
4. Triple intrathecal injection: d1, d43.

Maintenance therapy

There are 5 groups, each consisting of the following treatments:

1. ATRA 25-30 mg/m^2^.day, orally in 2-3 times, d1-21, d43-63.
2. Compound Realgar Natural Indigo Tablet 60 mg/kg/d, po, orally three times, d1-21.
3. 6MP 50 mg/m ^2^, QN per day, orally, for 8 weeks from d29.
4. MTX 25 mg/m^2^ QW, orally, for 8 weeks from d29.

Handling of poor responders and indications for hematopoietic stem cell transplantation

Two courses of consolidation therapy (IDA 8 mg/m^2^. D, QD, d1-3, 2 courses) are added to the low-risk patients whose *PML/RARA* is not negative or whose *PML/RARA* is positive for 2 consecutive times after turning negative after consolidation therapy and who are transferred to the high-risk group. The patients whose *PML/RARA* turn negative after add-on therapy continue the maintenance therapy for the high-risk group from the beginning. Patients whose *PML/RARA* cannot turn negative should be prepared for hematopoietic stem cell transplantation, and 2-3 rounds of post-remission treatment for the high-risk group should be given before transplantation. The high-risk patients whose *PML/RARA* is not negative or whose *PML/RARA* is positive 2 consecutive times after turning negative after consolidation therapy should be prepared for hematopoietic stem cell transplantation. The high-risk group consolidation therapy containing anthracycline and the high dose cytarabine regimen should be added before transplantation.

Figure. S1.


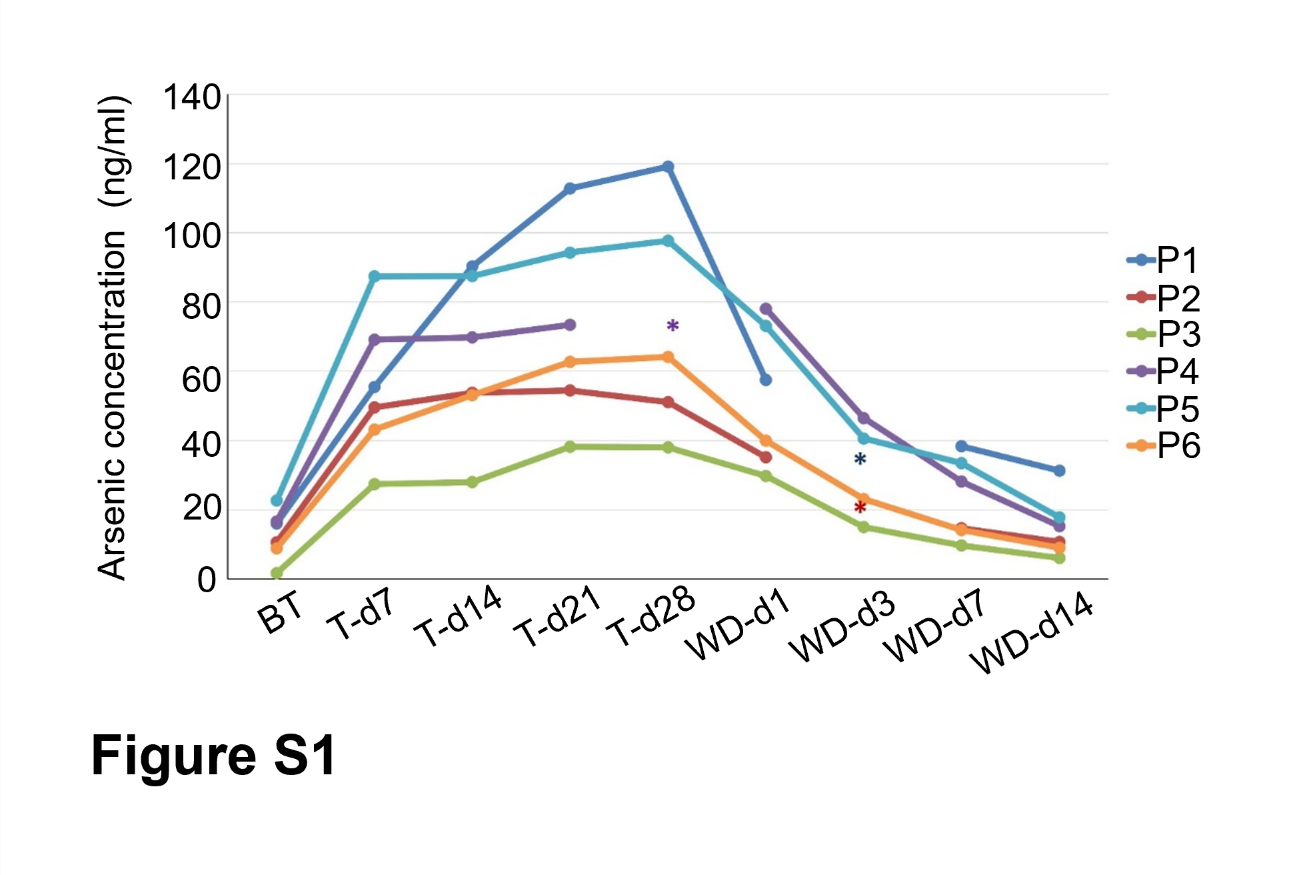


**Arsenic blood concentration in APL children**. We collected venous blood from six patients and tested the concentration of arsenic by inductively coupled plasma mass spectrometry. Subsequent measurements are taken at 7, 14, 21, and 28 days after commencing medication, as well as at 1, 3, 7, and 14 days after discontinuation. P1-P6, patient 1-6; BT, before taking RIF; T-d7, d14, d21, d28: 7, 14, 21, 28 days after taking RIF; WD-d1, d3, d7, d14: 1, 3, 7, 14 days after withdrawing RIF.

Figure. S2.


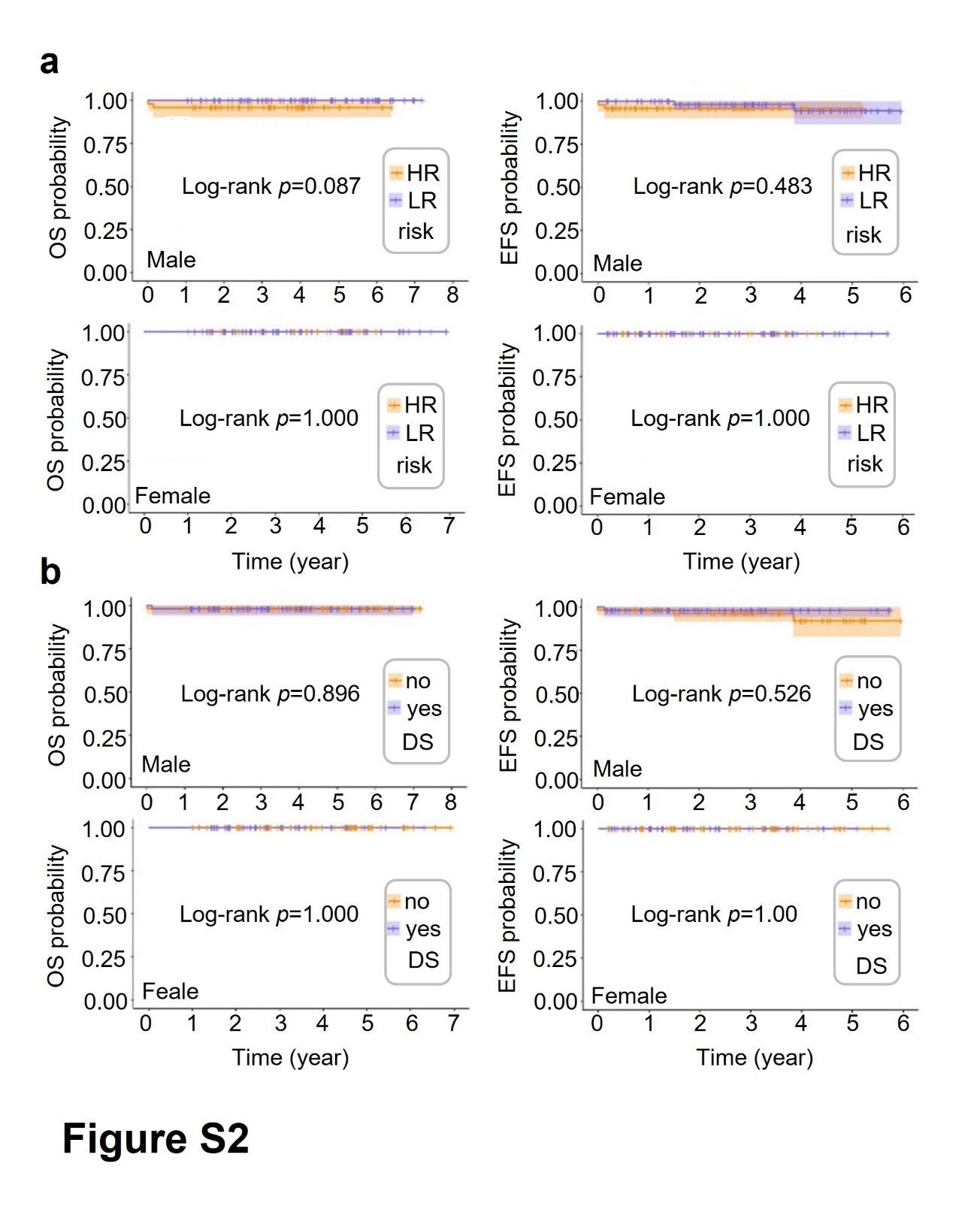


**Survival analysis in gender subgroup**. **a** We performed the OS prognosis analyses for the patients in different groups of risk (HR/LR) or DS (yes/no) in the subgroups of males and females. **b** EFS was similarly analyzed within these gender-specific subgroups. The Kaplan-Meier survival curves with *p* value were provided. OS, overall survival; EFS, event-free survival; HR, high-risk; LR, low-risk; DS, differentiation syndrome.

Figure. S3.


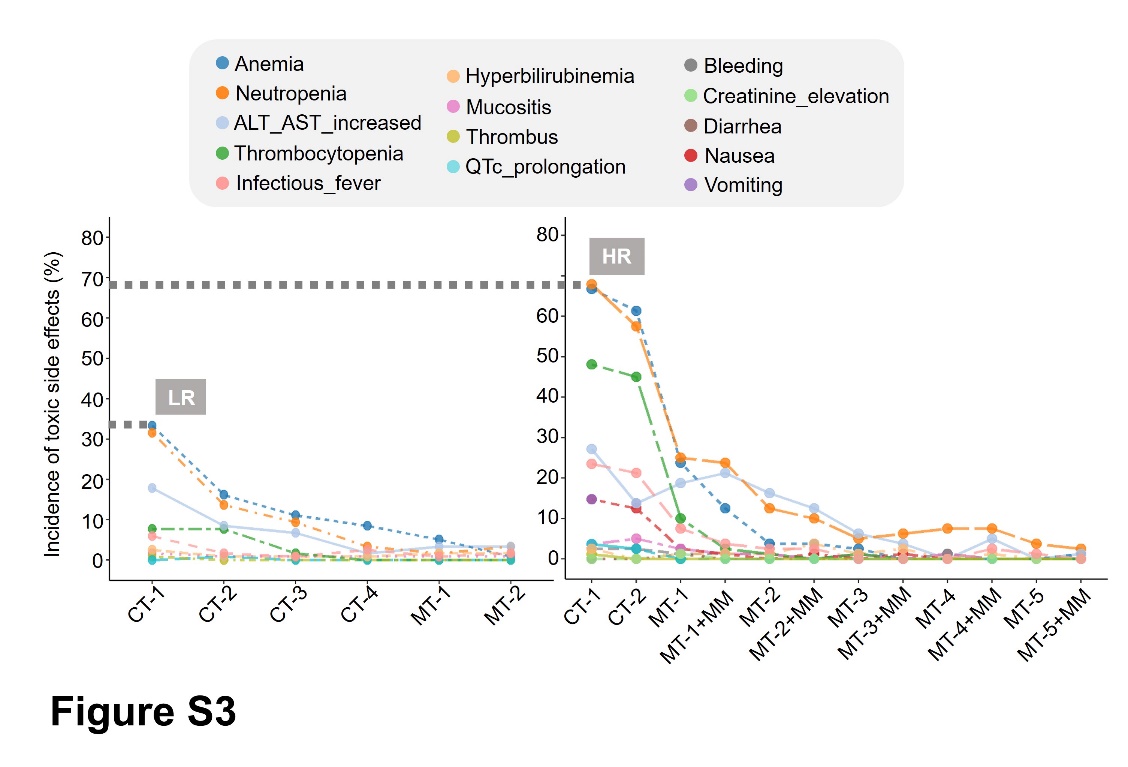


**Treatment toxicities during consolidation therapy and maintenance treatment of LR/HR.** We plotted the proportion of drug toxicity occurring at different stages of treatment in HR and LR groups. HR, high-risk; LR, low-risk; CT, consolidation treatment; MT, maintenance treatment without mercaptopurine and methotrexate; MT-MM: maintenance treatment with mercaptopurine and methotrexate; QTc, corrected QT interval; ALT/AST, alanine transaminase / aspartate aminotransferase.

Figure. S4.


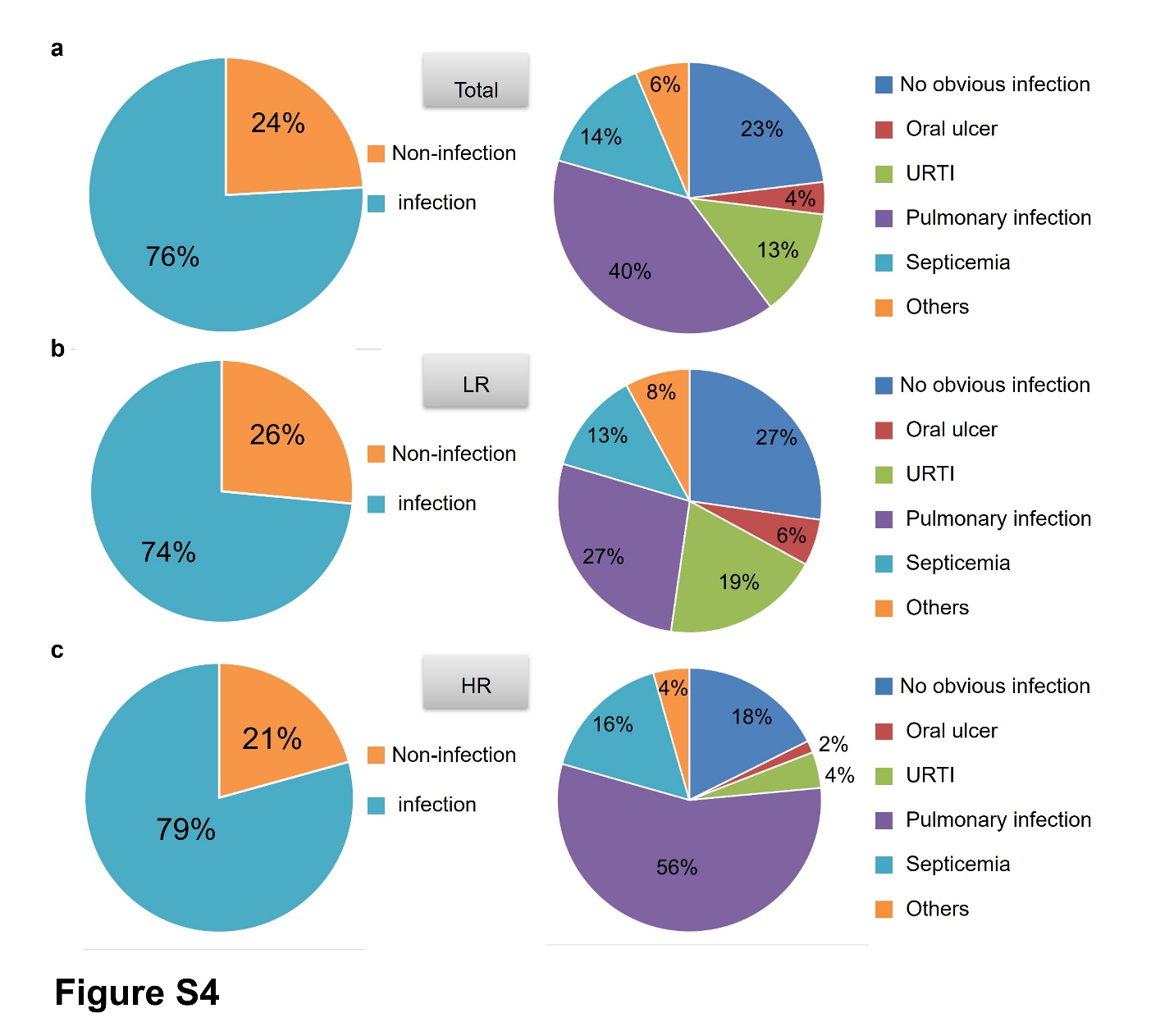


**Infection status during induction therapy**. **a** We first analyzed the infection status in the total patient cohort, categorizing patients into infection and non-infection groups, and further detailing the proportions of different types of infections. **b,c** We then stratified the patients into LR and HR subgroups and conducted a similar analysis within each subgroup to assess the infection status. The results are presented in pie charts, showing the distribution of infection and non-infection cases, as well as the specific types of infections in each subgroup. HR, high-risk; LR, low-risk; URTI, upper respiratory tract infection.

Figure. S5.


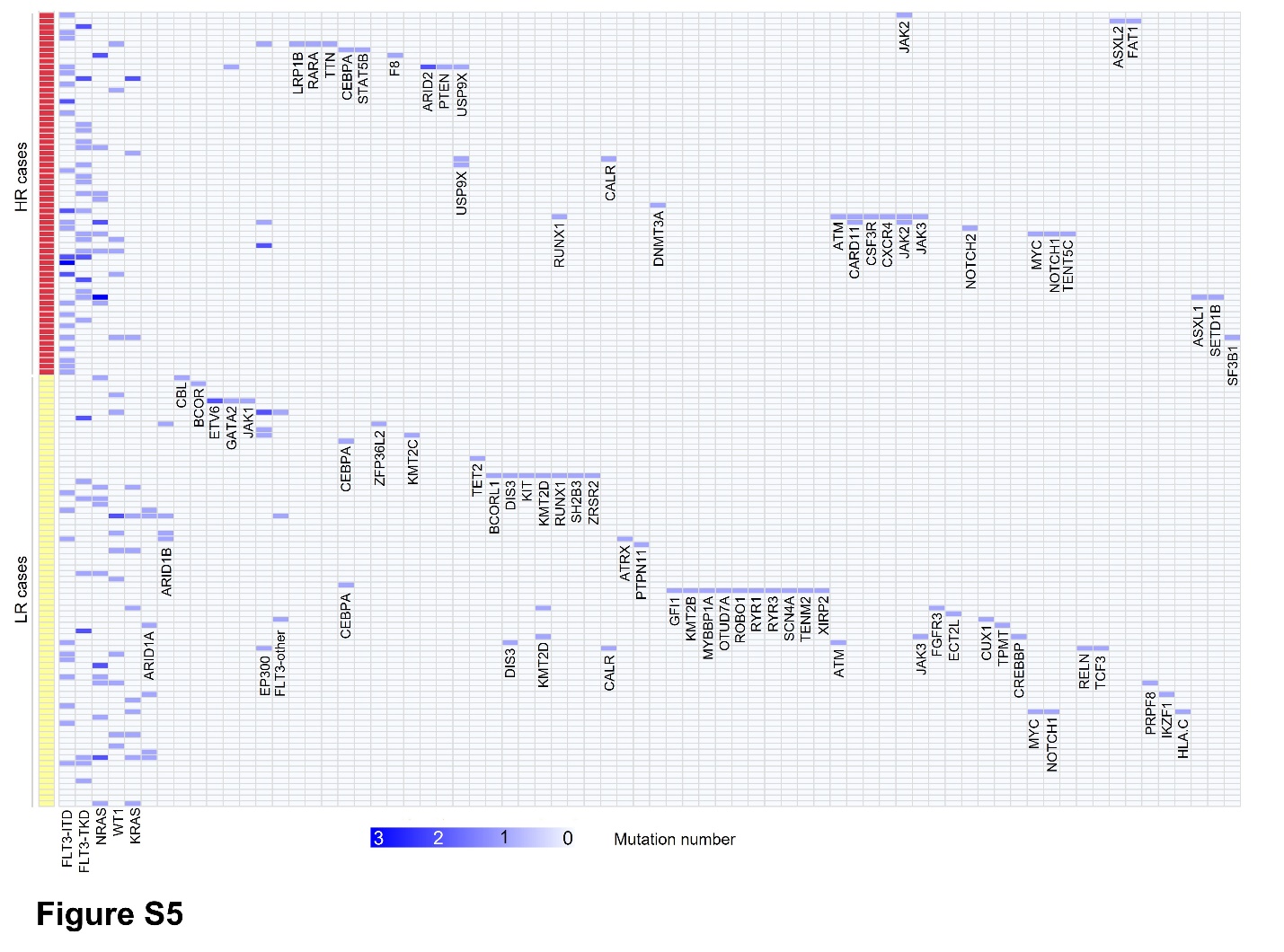


**A heat map illustrating gene mutations in children with APL.** We initially integrated the patients' risk profiles with gene mutation data, organized the patients according to their risk levels, and visualized the results using the pheatmap R package. The number of mutations is depicted in the figure.

Figure. S6.


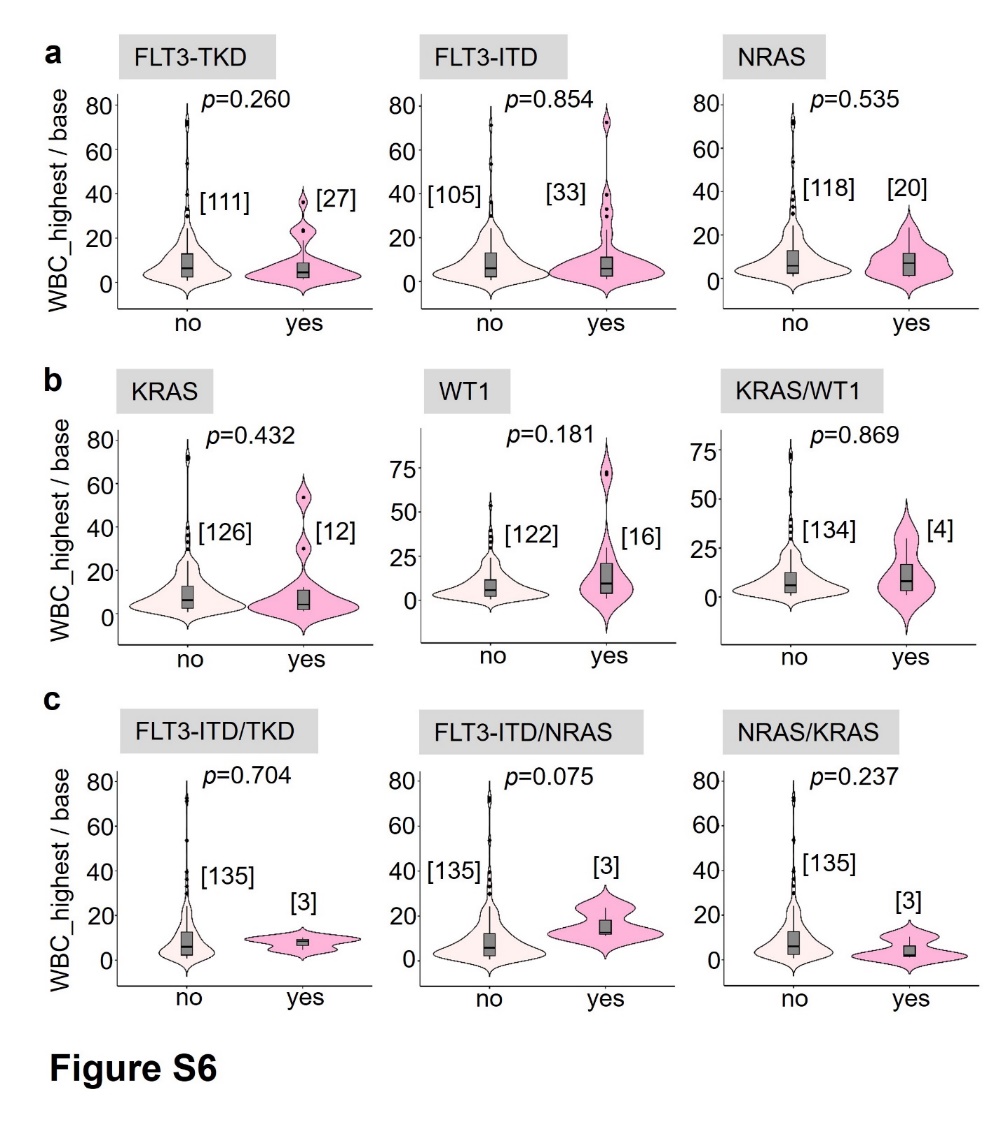


**Correlation between gene mutations and WBC changes during chemotherapy in APL patients**. For each APL patient, the WBC_highest/base ratio was calculated using the WBC_highest and WBC_base values and combined with single or dual gene mutation data. The sample size for each group was counted, and the Mann-Whitney U Test was used to compare the differences between groups. Visualization was performed using the ggviolin function in the ggpubr R package. **a** FLT3-TKD, FLT3-ITD, NRAS; **b** KRAS, WT1, KRAS/WT1; **c** FLT3-ITD/TKD, FLT3-ITD/NRAS, NRAS/KRAS. FLT3-TKD, FMS-like tyrosine kinase 3 - tyrosine kinase domain; FLT3-ITD, FMS-like tyrosine kinase 3 - internal tandem duplication; NRAS, Neuroblastoma RAS viral oncogene homolog; KRAS, Kirsten rat sarcoma viral oncogene homolog; WT1, Wilms tumor 1.

Figure. S7.


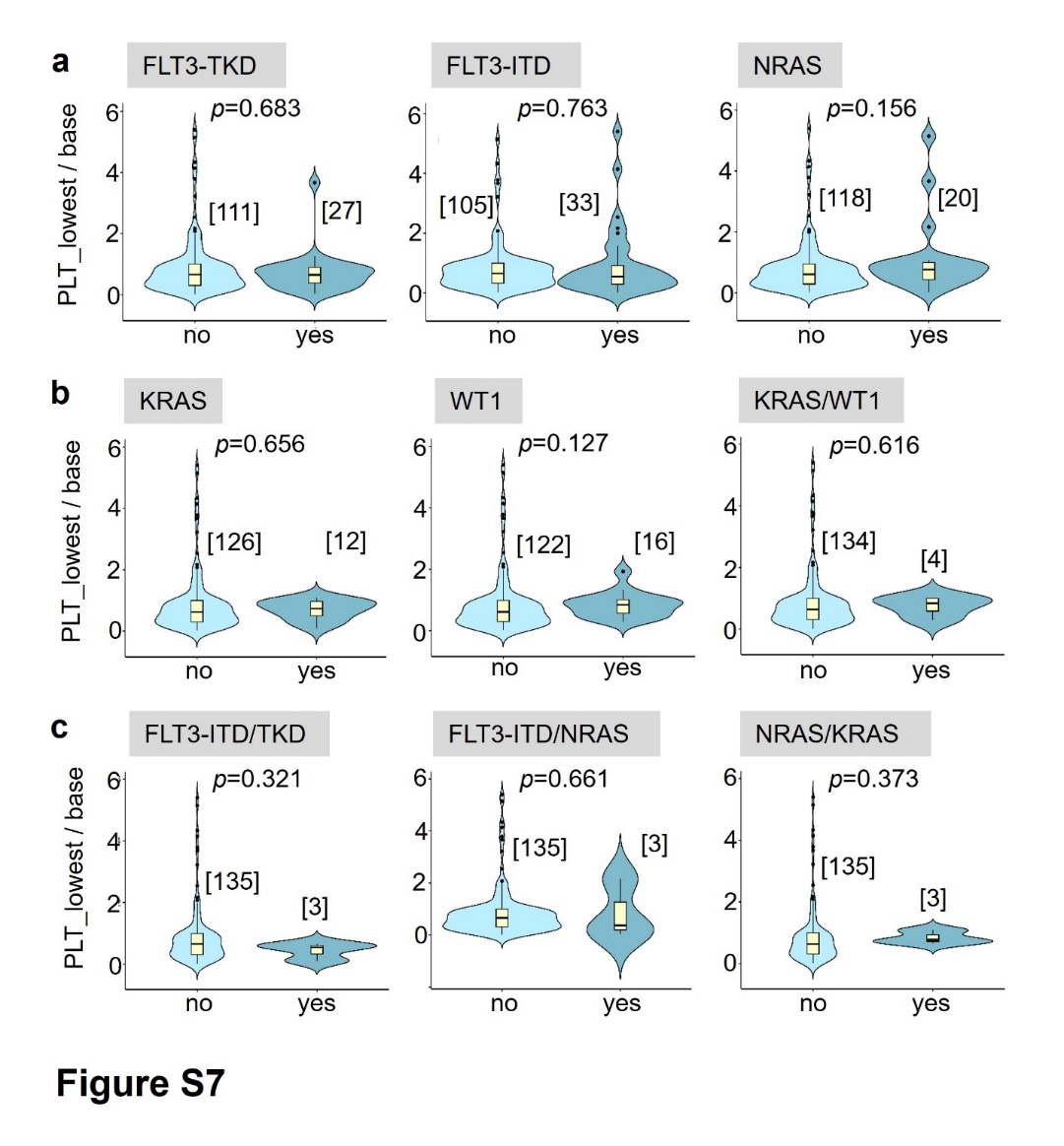


**Correlation between gene mutations and PLT changes during chemotherapy in APL patients.** For each APL patient, the PLT_lowest/base ratio was calculated using the PLT_lowest and PLT_base values and combined with single or dual gene mutation data. The sample size for each group was counted, and the Mann-Whitney U Test was used to compare the differences between groups. Visualization was performed using the ggviolin function in the ggpubr R package. **a** FLT3-TKD, FLT3-ITD, NRAS; **b** KRAS, WT1, KRAS/WT1; **c** FLT3-ITD/TKD, FLT3-ITD/NRAS, NRAS/KRAS. FLT3-TKD, FMS-like tyrosine kinase 3 - tyrosine kinase domain; FLT3-ITD, FMS-like tyrosine kinase 3 - internal tandem duplication; NRAS, Neuroblastoma RAS viral oncogene homolog; KRAS, Kirsten rat sarcoma viral oncogene homolog; WT1, Wilms tumor 1.

Figure. S8.


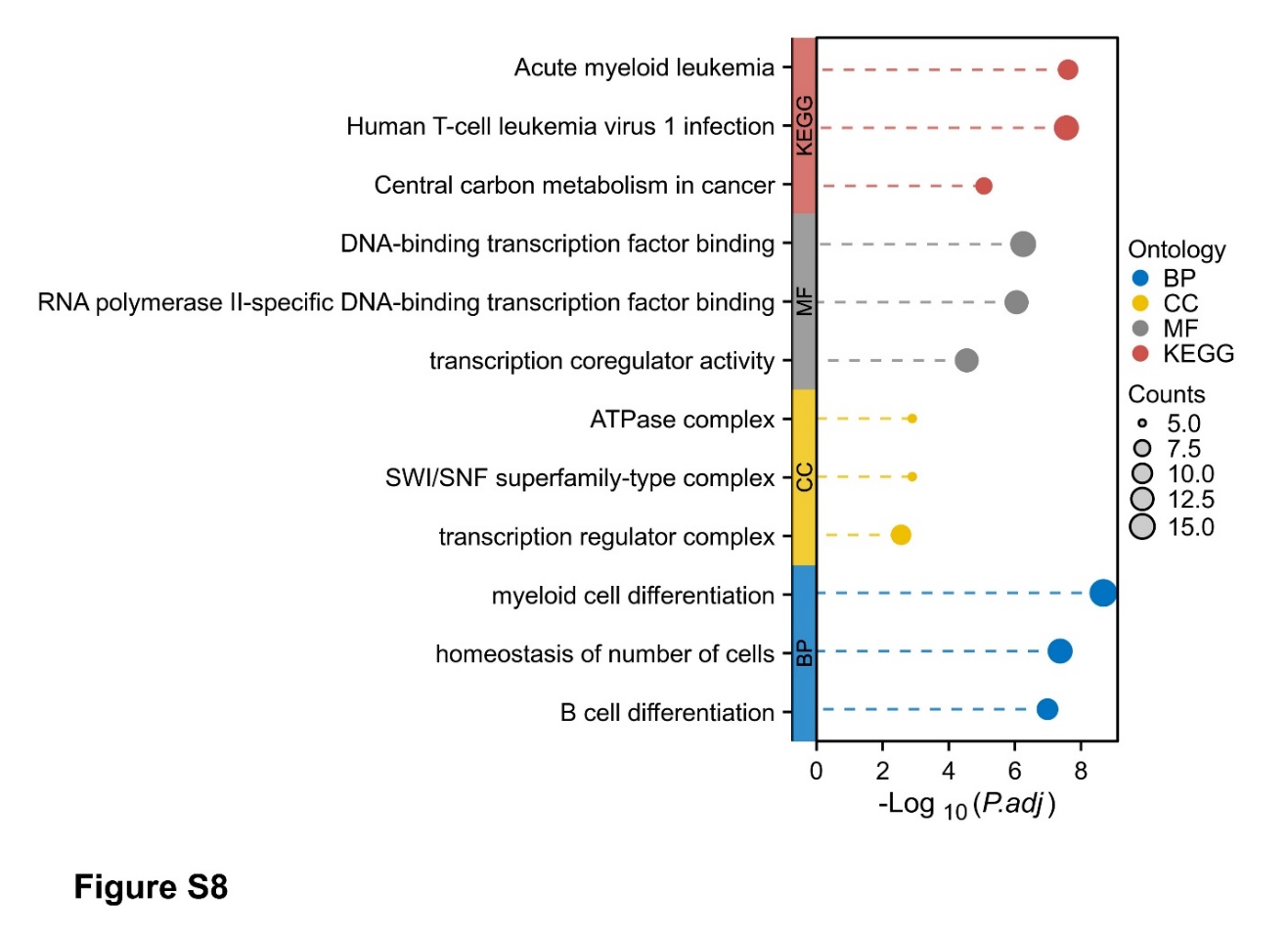


**Enrichment analysis of mutated genes based on GO and KEGG pathway annotation**. Gene identifiers were converted using the org.Hs.eg.db R package. Subsequent enrichment analysis was performed using the clusterProfiler R package, and the analysis results were visualized using the ggplot2 R package. GO, Gene Ontology; KEGG, Kyoto Encyclopedia of Genes and Genomes; BP, biological processes; CC, cellular components; MF, molecular functions.

Figure. S9.


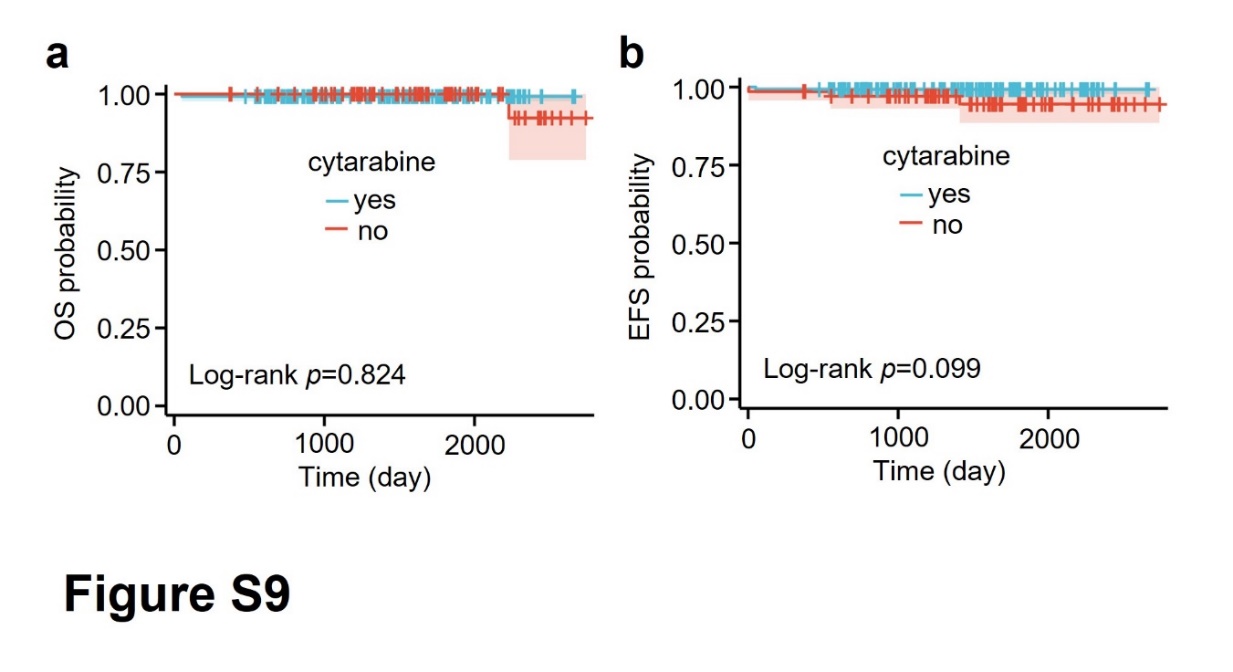


**Survival prognosis analysis of cytarabine. a** OS and **b** EFS were evaluated in patients stratified by cytarabine treatment status (yes/no). Kaplan-Meier survival curves are shown with corresponding *p* values from the Log-rank test.

Table S1.

### Arsenic blood concentration (ng/ml) in APL children.

| **Patients** | **BT** | **T-d7** | **T-d14** | **T-d21** | **T-d28** | **WD-d1** | **WD-d3** | **WD-d7** | **WD-d14** |
| --- | --- | --- | --- | --- | --- | --- | --- | --- | --- |
| **P1** | 16 | 55.5 | 90.3 | 112.8 | 119.1 | 57.5 | NC | 38.4 | 31.3 |
| **P2** | 10.6 | 49.6 | 53.8 | 54.5 | 51.1 | 35.2 | NC | 14.7 | 10.7 |
| **P3** | 1.66 | 27.4 | 28 | 38.2 | 38 | 29.8 | 15 | 9.68 | 6.05 |
| **P4** | 16.6 | 69.1 | 69.8 | 73.4 | NC | 78.1 | 46.5 | 28.2 | 15.3 |
| **P5** | 22.7 | 87.4 | 87.5 | 94.3 | 97.7 | 73.1 | 40.6 | 33.5 | 17.8 |
| **P6** | 8.86 | 43.2 | 53.1 | 62.7 | 64.2 | 40 | 23.1 | 14.2 | 9.02 |
| **Mean** | 12.7 | 55.4 | 63.8 | 72.7 | 74.0 | 52.3 | 31.3 | 23.1 | 15.0 |
| **median (IQR)** | 13.3 (9.3,16.5) | 52.5 (44.8,65.7) | 61.8 (53.3,83.1) | 68.1 (56.5,89.1) | 64.2 (51.1,97.7) | 48.8 (36.4,69.2) | 31.9 (21.1,42.1) | 21.4 (14.3,32.2) | 13 (9.4,17.2) |
| % (>45.7) | 0 | 66.7 | 83.3 | 83.3 | 80 | 50 | 25 | 0 | 0 |

APL, acute promyelocytic leukemia; P1-P6, patient 1-6; BT, before taking RIF;

T-d7, d14, d21, d28: 7, 14, 21, 28 days after taking RIF;

WD-d1, d3, d7, d14: 1, 3, 7, 14 days after withdrawing RIF;

NC, no concentration was measured; IQR, interquartile range (Q1, Q3).

Table S2.

### *PML/RARA* fusion gene and chromosome karyotype in APL children.

| **Risk** | **Qualitative of *PML/RARA*** | **Quantification (%) or copy number of *PML/RARA*** | **Karyotype** |
| --- | --- | --- | --- |
| **LR** | positive | 115.63% | 46,XX,t(15;17)(q22;q21)[2]/46,XX[1] |
| **LR** | positive | 48.17% | 46,XY,t(15;17)9q22;q21)[18]/46,XY[2] |
| **LR** | positive | UN | 46,XY,t(15;17)9q22;q21)[15]/46,XY[5] |
| **HR** | positive | UN | 46,XY,t(15;17)(q22;q21)[20] |
| **LR** | positive | 106.38% | 46,XY,der(15)t(15;17)(q22;q21),ider(17)(q10)t(15;17)[9]  /46,idem,t(1;4)(q32;q35),  ?del(11)(q23)[cp4]/46,XY[7] |
| **HR** | positive | UN | 46,XY[2] |
| **LR** | positive | 10.41% | 46,XY,t(15;17)(q24;q21)[17]/46,XY[3] |
| **LR** | positive | 57.97% | 46,XY,t(15;17)(q24;q21)[1]/46,XY[1] |
| **LR** | positive | UN | 46,XX,t(15;17)(q24;q21)[16]/46,XX[4] |
| **HR** | positive | UN | 46,XX,t(15;17)(q24;q21)[3]/46,XX[8] |
| **HR** | positive | 101.45% | 46,XY[20] |
| **LR** | positive | UN | 46,XY,t(15;17)(q24;q21)[15]/46,XY[5] |
| **LR** | positive | 46.06% | 46,XY,-6,t(15;17)(q24;q21),+mar[4] |
| **LR** | positive | UN | 45,XY,t(15;17)(q24;q21),-18[1]/46,XY[11] |
| **LR** | positive | UN | 46,XY,t(15;17)(q24;q21)[18]/46,XY[2] |
| **HR** | positive | UN | 46,XY,t(15;17)(q24;q21)[20] |
| **LR** | positive | 20.45% | 46,XY,t(15;17)(q24;q21)[8]/46,XY[2] |
| **LR** | positive | UN | 46,XX[3] |
| **LR** | positive | 38.82% | 46,XX,t(15;17)(q24;q21)[20] |
| **LR** | positive | 100.12% | 46,XX,t(15;17)(q24;q21)[15]/46,XX[5] |
| **HR** | positive | 91.17% | 46,XX[14] |
| **LR** | positive | 51.01% | 46,XY,t(15;17)(q24;q21)[20] |
| **HR** | positive | UN | 46,XY,t(15;17)(q24;q21)[10]/46,XY[10] |
| **LR** | positive | 33.9% | 46,XX,der(15)t(15;17)(q24;q21),-17,+mar[1]/46,idem,  add(1)(p34)[1]/46,XX,add(1)(p34),t(15;17)[1]/46,XX[17] |
| **HR** | positive | 35.05% | 46,XX,t(15;17)(q24;q21)[20] |
| **HR** | positive | 66.89% | 46,XY,t(15;17)(q24;q21)[10]/46,XY[10] |
| **LR** | positive | 48.51% | 46,XX,t(15;17)(q24;q21)[10]/46,XX[5] |
| **LR** | positive | 115.74% | 46,XX,t(15;17)(q24;q21)[20] |
| **LR** | positive | 57.69% | 46,XY,t(15;17)(q24;q21)[6]/46,XY[14] |
| **LR** | positive | 84.184% | 89,XXYY,-3,-9,t(15;17)(q24;q21),t(15;17)(q24;q21),-19[5]/46,XY[4] |
| **HR** | positive | 65.731% | 46,XY,t(15;17)(q24;q21)[19]/46,XY[1] |
| **HR** | positive | 18.346% | 46,XY,t(15;17)(q24;q21)[5] |
| **HR** | positive | 44.424% | 46,XY,t(15;17)(q24;q21)[15]/46,XY[5] |
| **LR** | positive | 89.432% | 46,XY,der(15)t(15;17)(q24;q21),ider(17)  (q10)t(15;17)(q24;q21)[14]/46,XY[6] |
| **LR** | positive | 33.112% | 46,XX,t(15;17)(q24;q21)[5]/46,idem,-6,+mar,inc[15] |
| **LR** | positive | UN | 46,XY[5] |
| **LR** | positive | 31.056% | 46,XX,t(15;17)(q24;q21)[16]/46,XX[4] |
| **LR** | positive | 11.531% | 46,XX[3] |
| **LR** | positive | 121.815% | 46,X,del(X)(q24),t(2;12)(p13;q24.3),t(6;14)(p21;q32),  t(15;17)(q24;q21)[17]/46,XX[3] |
| **LR** | positive | 35.989% | 46,XY,t(15;17)(q24;q21)[1]/47,idem,+mar[2]/46,XY[7] |
| **HR** | positive | 41.192% | 46,XX,t(15;17)(q24;q21)[20] |
| **HR** | positive | 56.143% | 46,XY,t(15;17)(q24;q21)[14]/46,XY[6] |
| **LR** | positive | 24.607% | 46,XY,t(15;17)(q24;q21)[20] |
| **LR** | positive | 34.163% | ND |
| **LR** | positive | 16.15% | 46,XX,t(15;17)(q24;q21)[14]/46,XX[6] |
| **HR** | positive | 6.54% | 46,XX,t(15;17)(q24;q21)[20] |
| **HR** | positive | 21.864% | 46,XX,t(15;17)(q24;q21)[19]/46,XX[1] |
| **HR** | positive | 132.089% | 46,XY,t(15;17)(q24;q21)[20] |
| **LR** | positive | 21.592% | 46,XX,t(15;17)(q24;q21)[2]/46,idem,der(7)t(7;13)(q31;q14)  [10]/46,idem,der(16)add(16)  (p13.3)del(16)(q2?2q24)[3]/46,idem,der(7)t(7;13),der(16)  add(16)(p13.3)del(16)(q2?2q24)[1]/46,XX[4] |
| **HR** | positive | 26.364% | 46,XX,t(15;17)(q24;q21)[18]/46,XX,del(13)(q12q14),  t(15;17)(q24;q21)[1]/46,XX[1] |
| **LR** | positive | 45.462% | 46,XY,t(15;17)(q24;q21)[20] |
| **HR** | positive | 27.569% | 46,XY,t(15;17)(q24;q21)[8]/46,XY[8] |
| **LR** | positive | 63.262% | 46,XX,t(1;14)(p22;q24),del(2)(q33),add(4)(q31),  t(15;17)(q24;q21)[4]/46,XX[16] |
| **LR** | positive | 69.666% | 46,XX,t(15;17)(q24;q21)[5]/46,XX,der(15)t(15;17),  ider(17)(q10)t(15;17)[9]/46,XX[6] |
| **HR** | positive | 37.99% | 46,XX,del(17)(p13)[6]/46,XX[14] |
| **LR** | positive | 38.414% | 46,XY,der(7)t(7;8)(q22;q13),t(15;17)(q24;q21)[20] |
| **HR** | positive | 37.134% | 46,XY,t(12;15;17)(p13;q24;q21)[11]/47,idem,+8[4]/47,  idem,del(9)(q13q32)[3]/47,idem,+mar[2] |
| **LR** | positive | UN | UN |
| **HR** | positive | UN | UN |
| **LR** | positive | UN | 46,XX,?Del(6)(p22),t(15;17)(q24;q21)[19]/46,XX[1] |
| **HR** | positive | UN | UN |
| **LR** | positive | UN | 46,XX,t(15,17)(q24,q21)[4] |
| **LR** | positive | UN | 46,XX,add(1)(q32),add(5)(q31),der(8)ins(8;?)(q22;?),  t(15;17)(q22;q21)[cp13],46,XX[7] |
| **LR** | positive | UN | 46,XX,t(15;17)(q24;q21)[19]/46,XX[1] |
| **HR** | positive | UN | 46,XX,t(15;17)(q22;q21)[12] |
| **LR** | positive | UN | UN |
| **HR** | positive | UN | ND |
| **LR** | positive | L41898912/V63798920/S6937401 | 46,XY,t(15;17)(q22;q21)[15]/46,XY[5] |
| **LR** | positive | L1506372720/V141400624/S47938792 | 46,XY,t(15;17)(q22;q21)[8]/46,XY[12] |
| **LR** | positive | UN | 46,XX,t(15;17)(q22,q21)[7]/46,XX[13] |
| **LR** | positive | V211732/S53916808 | 46,XY,t(15;17)(q22;q21)[10]/49,del(6)(q12;q21),+add(19)(q12)*3[4]/46,XX[6] |
| **HR** | positive | UN | 46,XY,t(2;17)(p12;q25)[16]/46,XY[4] |
| **HR** | positive | s119703128 | 46,XX,t(15;17)(q22;q21),der(20)t(8;20)(q13;q13.3)/46,XX |
| **LR** | positive | s83810920 | 46,XX[20] |
| **HR** | positive | s8369476 | 46,XX,t(15;17)(q22;q21)/46,XX |
| **LR** | positive | UN | UN |
| **HR** | positive | s75980504 | 46,XX,t(15;17)(q22;q21) |
| **LR** | positive | L20615252/v30643424/s33704672 | 46,XX |
| **LR** | positive | L984225/V3707765/S2038302 | 46,XY,t(15;17)(q22;q21) |
| **LR** | positive | L2468715/V4551025/S4754340 | 46,XY,t(15;17)(q22;q21) |
| **LR** | positive | L38209 | 46,XX,t(15;17)(q22;q21) |
| **LR** | positive | V2779065/S895292 | 46,XY,t(15;17)(q22;q21) |
| **LR** | positive | L6501858/V1007326/S2470954 | 46,XY,t(15;17)(q22;q21) |
| **LR** | positive | L1257073/V2541019/S1388610 | 46,XX,t(15;17)(q22;q21) |
| **LR** | positive | L1905295/V3601835/S1057479 | 46,XX,t(15;17)(q22;q21) |
| **LR** | positive | L60789572/V14794046/S46761564 | 46,XX,t(15;17)(q22;q21) |
| **LR** | positive | UN | UN |
| **LR** | positive | L1671048/V40693384/S7979227 | 46,XY,t(15;17)(q22;q21)/47,idem,+8 |
| **HR** | positive | L4750982/V5720623/S293196 | 46,XY,t(15;17)(q22;q21) |
| **LR** | positive | L50367100/V18455850/S5444760 | 46,XY,der(15)t(15;17)(q22;q21),ider(17)(q10)t(15;17)(q22;q21) |
| **LR** | positive | L63093416/V12317840/S6208946 | 46,XY,t(15;17)(q22;q21) |
| **LR** | positive | S4786158 | 46,XY,der(15)t(15;17)(q22;q21),ider(17)(q10)t(15;17)(q22;q21)/46,XY |
| **HR** | positive | S8073217 | 46,XX,t(15;17)(q22;q21) |
| **HR** | positive | S6996338 | 46,XY,t(15;17)(q22;q21) |
| **HR** | positive | 83665784 | 46,XX,t(15;17)(q22;q21) |
| **LR** | positive | L4511187/V21113254/S5034997 | UN |
| **LR** | positive | V220000 | 46,XY,i(17)(q10) |
| **LR** | positive | UN | 46,XX,t(5;22)(q13;q11),t(15;17;17)(q22;q25;q21)[4]/46,  idem,t(8;19)(p21;q13)[5]/46,XX[1] |
| **LR** | positive | UN | 46,XY,t(15;17)(q22;q21)[19]/46,XY[1] |
| **HR** | positive | UN | 46,XY,t(15;17)(q22;q21)[11]/46,XY[4] |
| **HR** | positive | UN | 46,XY,t(15;17)(q22;q21)[5] |
| **HR** | positive | UN | 46,XX,t(15;17)(q22;q21)[10] |
| **HR** | positive | UN | 46,XX,t(15;17)(q22;q21)[14]/46,XX[6] |
| **LR** | positive | UN | 46,XX,t(15;17)(q22;q21)[20] |
| **LR** | positive | UN | 46,XY,t(15;17)(q22;q21)[2]/46,XY[1] |
| **LR** | positive | UN | 46,XY,t(15;17)(q22;q21)[6] |
| **LR** | positive | UN | 46,XY,t(15;17)(q22;q21)[10] |
| **LR** | positive | UN | 46,XX,t(15;17)(q22;q21)[8]/46,XX[2] |
| **LR** | positive | 3801001 | 46,XX,t(15;17)(q22;q21)[20] |
| **HR** | positive | 2482963 | 46,XX,t(15;17)(q22;q21)[20] |
| **HR** | positive | 1095787 | 46,XX,t(15;17)(q22;q21)[20] |
| **LR** | positive | 655623 | 46,XX,t(15;17)(q24;q21)[20] |
| **LR** | positive | 119.72% | 46,XY,t(15,17)(q24;q21)[15]/46,XY[5] |
| **LR** | positive | UN | 46,XY,t(10;13)(q23;q26)(?),der(15)t(15,17)(q24;q21)t(15;17)(q23;q26)(?),-16,der(17)t(15;17)(q24;q21)t(15;17)(q23;q26)(?),+mar[17]/46,XY[6] |
| **LR** | positive | UN | 46,XX,t(15,17)(q22;q21)[20] |
| **LR** | positive | UN | 46,XY,t(15,17)(q24;p13)[15]/46,XY[5] |
| **HR** | positive | 55.97% | 46,XX,t(15;17)(q24;q21)[10]/46,XX[1] |
| **LR** | positive | 50.7% | 47,XX,der(15)t(15;17)(q24;q21),der(17),+mar[19]/46,XX[1] |
| **HR** | positive | 31639 | 46,XY[20] |
| **HR** | positive | UN | 46,XX[2] |
| **LR** | positive | UN | 46,XX[18] |
| **HR** | positive | 5316 | 46, XY, t(15;17) (q22: q21) [17]/47, idem,+8[3] |
| **LR** | positive | 1.8*10 e5(0.517 relative to ABL1) | 46,XY[4] |
| **LR** | positive | 1.53*10 e3(0.0404 relative to ABL1) | 46,XY,t(15;17) |
| **LR** | positive | ND | 46,XY, t(15;17)(q22;q21)[20] |
| **LR** | positive | ND | 46,XY, t(15;17)(q22;q21)[18]/46, XY [2] |
| **LR** | positive | ND | 46,XY, t(15;17)(q22;q21)[20] |
| **LR** | positive | ND | 46,XY, t(15;17)(q22;q21)[3]/46.idem,add(X)(p22.1),  -2,add(7)(q22),-22,+mar1,+mar2[15]/46,XX[2] |
| **LR** | positive | ND | 46,XY,t(15;17)(q22;q21)[20] |
| **LR** | positive | ND | 46,XY, t(15;17)(q22;q21)[20] |
| **LR** | positive | ND | 46,XY, t(15;17)(q22;q21)[17]/46, XY [3] |
| **HR** | positive | 241736 | 46,XX,-?15,add(17)(q25),del(22)(q11),+mar,inc[8] |
| **HR** | positive | ND | 48,XY,+8,+8,t(15;17)(q22;q21)[5]/46,XY[3] |
| **HR** | positive | 32204 | 46,XX,t(15;17)(q22;q21)[19]/46,XX[1] |
| **HR** | positive | 5630 | 46,XY,der(15)t(15;17)(q22;q21),ider(17)(q10)t(15;17)  [10]/45,idem,-Y,t(1;2)(p13;q33)[10] |
| **HR** | positive | 5112 | 46,XY,t(15;17)(q22;q21)[20] |
| **HR** | positive | 121091 | 46,XY,t(15;17)(q22;q21) |
| **LR** | positive | 5848 | 46,XX,t(15;17;17)(q22;q25;q21)[14]/46,idem,?  add(X)(q22),-2,del(9)(q22),  -16,-21,+mar1,+mar2,+mar3[6] |
| **LR** | positive | 56934 | 46,XX,t(15:17)(q22:q21)/46,XX |
| **HR** | positive | 7300 | 46,XX,t(15;17)(q22;q21) |
| **LR** | positive | 20849 | 46,XY,add(7)(q22),t(15;17)(q22;q21)[11]/46,XY[5] |
| **HR** | positive | 3798 | 46,XX,t(15;17)(q22;q21)[19]/46,XX[1] |
| **LR** | positive | 25519 | 47,XY,+4[17]/46,XY |
| **LR** | positive | UN | 46,XY,t(15;17)(q22;q21)「3」/46,XY(7) |
| **LR** | positive | 70.4% | 46,XY,t(15;17)(q22;q21)[9]/46,XY[1]; |
| **LR** | positive | UN | 46,XY,t(15;17)(q22;q21)[11]) |
| **HR** | positive | UN | 46,XY,t(15;17)(q22;q21) |
| **LR** | positive | UN | 46,XX,t(15;17)(q22;q21) |
| **LR** | positive | 8.30% | 46,XY,t(15:17)(q22;q21) |
| **HR** | positive | UN | 46,XX,t(15;17)(q22;q21)[2]/46,IDEM,-6,+r[6]/46,XX,  der(15)t(15;17)(q22;q21),ider(17)(q10)t(15;17)[2] |
| **HR** | positive | UN | 46,XY,t(15;17)(q22;q21) |
| **HR** | positive | UN | 46XY,t(15;17)(q22;q21)。 |
| **HR** | positive | UN | 46,XY,t(15;17)(q22;q21)[19]/46,XY[1] |
| **LR** | positive | UN | 46,XY[4] |
| **LR** | positive | UN | 46,XY,t(15,17)(q22,q21)[18]/46,XY,der(15)t(15;17),  ider(17)(q10)t(15;17)[2] |
| **LR** | positive | 145.13% | 46,XX,t(15;17)(q22;q21) |
| **LR** | positive | 26.94% | 46,XY,t(15;17)(q22;q21)[20] |
| **HR** | positive | 17.82% | 46,XY,t(15;17)(q22;q21)[20], |
| **HR** | positive | 29.06% | 46,XX,t(15;17)(q22;q21)[12]/46,XX[15] |
| **HR** | positive | 21.79% | 46,XY,t(15;17)(q22;q21)[13]/46,XY[3] |
| **LR** | positive | UN | 46,XY,t(15;17)(q22;q21)[17]/46,XY[3] |
| **HR** | positive | 39.93% | 46,XY |
| **HR** | positive | 33.50% | ND |
| **HR** | positive | 38.02% | 46,XY,t(15;17)(q22;q21) |
| **HR** | positive | 22.34% | 46,XY |
| **HR** | positive | UN | ND |
| **LR** | positive | 10.60% | 46,XX,t(15;17)(q22;q21)[20] |
| **HR** | positive | 14.83% | 46,XY,t(15;17)(q22;q21) |
| **HR** | positive | 38.36% | 46,XY,t(15;17)(q22;q21)[18]/46,XY,[2] |
| **HR** | positive | 9.19% | 46,XY,t(15;17)(q22;q21)[6]/46,XY,[14] |
| **HR** | positive | UN | 46,XY,t(15;17)(q22;q21) |
| **LR** | positive | 27.82% | 46,XY,add(7)(q32),t(15;17)(q22;q21)[20] |
| **LR** | positive | 51.30% | 46,XX,T(15;17)(q22;q21)[8]/46,XY[3] |
| **LR** | positive | 1.12% | 46,XY,t(15;17)(q22;q21)[19]/46,XY[1] |
| **HR** | positive | UN | 45,X,-X,t(15;17)(q22;q21)[19]/46,XX[1] |
| **HR** | positive | 2.78% | 46,XX[20] |
| **LR** | positive | 11.64% | 46,XX,t(15;17)(q24;q21)[19]/46,XX[1] |
| **HR** | positive | 27% | 46,XY,del(7)(q？31),t(15;17)  (q24;q21)[18]/46,XY,add(7)(q32),t(15;17)(q24;q21) |
| **HR** | positive | 16.68% | 46,XX,t(15,17)(q24;q21)[20] |
| **HR** | positive | 13.39% | 46,XY,t(1;17;15)(p22;q21;24)[20] |
| **HR** | positive | 29.40% | 46,XY,t(25,;17)(q22;q21)[16]/46,XY[4] |
| **HR** | positive | 27.43% | 46,XY,der(15)t(15;17)(q22;q21),ider(17)(q10) t(15;17)[20] |
| **HR** | positive | 38.56% | 46,XX,?add(17)(p11)[11]/46,XX[4] |
| **LR** | positive | 12.40% | 46,XX,t(15;17)(q24;q21)[12]/48,idem,+mar1~2[8] |
| **HR** | positive | 10.83% | 92,XXYY,t(15;17)(q24;q21)x2(6) |
| **LR** | positive | 11.14% | 46,XY,t(15;17)(q24;q21)[18]/46,XY[2] |
| **LR** | positive | 28% | 46,XX,add(70(q36)[15]/46,XX[5] |
| **HR** | positive | 21.42% | 46,XY,der(15)t(15;17)(q22;q21),ider(17)(q10)t(15;17)[20] |
| **LR** | positive | 22.25% | 46,XY,t(15;17)(q24;q21)[16]/46,XY[4] |
| **LR** | positive | 9.48% | 46,XY,t(15;17)(q24;q21)[20] |
| **HR** | positive | 10.90% | 46,XY,t(15;17)(q24;q21)[19]/46,XY[1] |
| **LR** | positive | 15.13% | 46,XY,t(15;17)(q24;q21)[18]/46,XY,[2] |
| **HR** | positive | 14.92% | 46,XY,t(15;17)(q24;q21)[7]/46,idem,?add(6)(p21)[5]/46,XY[8] |
| **LR** | positive | 11.13% | 46,XY,der(17)t(15;17)(q22;q21)[7]/46,XY[13] |
| **HR** | positive | 19.07% | 46,XX,t(15;17)(q22;q21)[13]/46,XX[7] |
| **LR** | positive | 9.48% | 46,XX,der(15)t(15;17)(q24;q21),ider(17)(q10)t(15;17)[20] |
| **LR** | positive | 22.78% | 46,XX,t(15;17)(q24;q21)[15]/46,XX[5] |
| **HR** | positive | 8.17% | 46,XY,t(4;15)(q25;q22),del(5)(q31),add(17)9q21)[19]/46,XY[1] |

HR, high-risk; LR, low-risk; *PML/RARA*, promyelocytic leukemia/retinoic acid receptor alpha; UN, uncertainty; ND, no mitosis observed.

Table S3.

### Incidence of differentiation syndrome.

| **DS** | **LR** | **HR** | ***p* value** |
| --- | --- | --- | --- |
| **no** | 77 (65.8%) | 45 (54.9%) | 0.119 |
| **yes** | 40 (34.2%) | 37 (45.1%) |  |
| **Total** | 117 | 82 |  |

HR, high-risk; LR, low-risk;

DS, differentiation syndrome

Table S4.

### Analysis of risk factors for differentiation syndrome.

| **Characteristics** | **DS_no** | **DS_yes** | ***p* value** |
| --- | --- | --- | --- |
| **n** | 122 | 77 |  |
| **WBC (×10^9^L), median (IQR)** | 3.95 (2.28, 13.76) | 8.22 (2.9, 30.14) | 0.03 |
| **HB (g/L), median (IQR)** | 80.5 (65.25, 94) | 76 (64, 92) | 0.38 |
| **PLT (×10^9^L), median (IQR)** | 33 (19, 65.75) | 34 (15, 45) | 0.30 |
| **Whether to use heparin in induction therapy, n (%)** |  |  | 0.49 |
| no | 110 (90.2%) | 67 (87.0%) |  |
| yes | 12 (9.8%) | 10 (13.0%) |  |
| **Whether to use hydroxyurea in induction therapy, n (%)** |  |  | 0.66 |
| yes | 95 (77.9%) | 62 (80.5%) |  |
| no | 27 (22.1%) | 15 (19.5%) |  |
| **Maximum WBC count, median (IQR)** | 37.53 (19.38, 73.10) | 51.48 (38.9, 84.4) | 0.0008 |
| **The day of chemotherapy with highest WBC, mean ± SD** | 10.90 ± 5.13 | 9.32 ± 4.69 | 0.03 |
| **Minimum PLT count, median (IQR)** | 18 (10, 37.75) | 12 (6, 20) | 0.0003 |
| **The day of chemotherapy with lowest PLT, median (IQR)** | 11 (2, 17) | 14 (8, 20) | 0.009 |

DS, differentiation syndrome; WBC, white blood cell; HB, Hemoglobin; PLT, platelet; IQR, interquartile range; SD, standard deviation.

Table S5.

### Assessment of toxic side effects according to CTCAE 4.0 during induction treatment phase.

| **Characteristics** | **LR** | **HR** | ***p* value** |
| --- | --- | --- | --- |
| **n** | 117 | 82 |  |
| **Side effects associated with inducement therapy, n (%)** |  |  | 1 |
| no | 1 (0.9%) | 0 (0.0%) |  |
| yes | 116 (99.1%) | 82 (100%) |  |
| **Anemia (grades), n (%)** |  |  | 0.64 |
| 0 | 2 (1.7 %) | 0 (0.0%) |  |
| 1 | 1 (0.9%) | 2 (2.4%) |  |
| 2 | 8 (6.8%) | 7 (8.6%) |  |
| 3 | 100 (85.5%) | 68 (82.9%) |  |
| 4 | 6 (5.1%) | 5 (6.1%) |  |
| **Neutropenia (grades, ×10^9^/L), n (%)** |  |  | 0.38 |
| 0 | 2 (1.7%) | 3 (3.65%) |  |
| 1 | 1 (0.9%) | 3 (3.65%) |  |
| 2 | 4 (3.4%) | 5 (6.1%) |  |
| 3 | 35 (29.9%) | 19 (23.2%) |  |
| 4 | 75 (64.1%) | 52 (63.4%) |  |
| **Thrombocytopenia classification, n (%)** |  |  | 0.36 |
| 0 | 7 (6.0%) | 2 (2.4%) |  |
| 1 | 3 (2.6%) | 1 (1.2%) |  |
| 2 | 10 (8.5%) | 3 (3.7%) |  |
| 3 | 21 (17.9%) | 14 (17.1%) |  |
| 4 | 76 (65.0%) | 62 (75.6%) |  |
| **Nausea, n (%)** |  |  | 0.17 |
| 0 | 67 (57.3%) | 40 (48.8%) |  |
| 1 | 30 (25.6%) | 17 (20.7%) |  |
| 2 | 18 (15.4%) | 23 (28.1%) |  |
| 3 | 2 (1.7%) | 2 (2.4%) |  |
| **Vomiting, n (%)** |  |  | 0.84 |
| 0 | 81 (69.2%) | 58 (70.7%) |  |
| 1 | 29 (24.8%) | 19 (23.2%) |  |
| 2 | 6 (5.1%) | 5 (6.1%) |  |
| 3 | 1 (0.9%) | 0 (0.0%) |  |
| **Diarrhea, n (%)** |  |  | 0.37 |
| 0 | 115 (98.2%) | 77 (93.9%) |  |
| 1 | 1 (0.9%) | 2 (2.4%) |  |
| 2 | 1 (0.9%) | 2 (2.4%) |  |
| 3 | 0 (0%) | 1 (1.2%) |  |
| **Mucositis, n (%)** |  |  | 0.10 |
| 0 | 99 (84.6%) | 61 (74.4%) |  |
| 1 | 15 (12.8%) | 13 (15.9%) |  |
| 2 | 0 (0.0%) | 2 (2.4%) |  |
| 3 | 3 (2.6%) | 6 (7.3%) |  |
| **DIC, n (%)** |  |  | 0.32 |
| 0 | 75 (64.1%) | 50 (62.0%) |  |
| 1 | 20 (17.1%) | 8 (9.8%) |  |
| 2 | 9 (7.7%) | 7 (8.5%) |  |
| 3 | 9 (7.7%) | 12 (14.6%) |  |
| 4 | 4 (3.4%) | 4 (4.9%) |  |
| 5 | 0 (0.0%) | 1 (1.2%) |  |
| **Bleeding, n (%)** |  |  | 0.003 |
| 0 | 87 (74.3%) | 40 (48.8%) |  |
| 1 | 6 (5.1%) | 7 (8.5%) |  |
| 2 | 18 (15.4%) | 31 (37.8%) |  |
| 3 | 1 (0.9%) | 0 (0.0%) |  |
| 4 | 5 (4.3%) | 3 (3.7%) |  |
| 5 | 0 (0.0%) | 1 (1.2%) |  |
| **Thrombus, n (%)** |  |  | 0.47 |
| 0 | 115 (98.2%) | 80 (97.6%) |  |
| 1 | 1 (0.9%) | 0 (0%) |  |
| 2 | 1 (0.9%) | 2 (2.4 %) |  |
| **QTc prolongation, n (%)** |  |  | 0.46 |
| 0 | 111 (94.9%) | 76 (92.7%) |  |
| 1 | 6 (5.1%) | 5 (6.1%) |  |
| 2 | 0 (0.0%) | 1 (1.2%) |  |
| **ALT/AST increased, n (%)** |  |  | 0.76 |
| 0 | 55 (47.0%) | 36 (43.9%) |  |
| 1 | 48 (41.0%) | 36 (43.9%) |  |
| 2 | 7 (6.0%) | 3 (3.7%) |  |
| 3 | 7 (6.0%) | 7 (8.5%) |  |
| **Hyperbilirubinemia, n (%)** |  |  | 0.39 |
| 0 | 111 (94.9%) | 75 (91.5%) |  |
| 1 | 6 (5.1%) | 6 (7.3%) |  |
| 2 | 0 (0.0%) | 1 (1.2%) |  |
| **Sepsis, n (%)** |  |  | 0.30 |
| no | 109 (93.2%) | 73 (89.0%) |  |
| yes | 8 (6.8%) | 9 (11.0%) |  |

HR, high-risk; LR, low-risk; CTCAE, common terminology criteria for adverse events;

DIC, disseminated intravascular coagulation;

QTc, corrected QT interval; ALT/AST, alanine transaminase / aspartate aminotransferase.

Table S6.

### Assessment of toxic side effects according to CTCAE 4.0 during consolidation and maintenance treatment phase in LR group.

| **Characteristics** | **CT-1 (n, %)** | **CT-2 (n, %)** | **CT-3 (n, %)** | **CT-4 (n, %)** | **MT-1 (n, %)** | **MT-2 (n, %)** | ***p* value** |
| --- | --- | --- | --- | --- | --- | --- | --- |
| **Anemia** |  |  |  |  |  |  | <0.001 |
| 0 | 78 (66.7%) | 98 (83.8%) | 104 (88.9%) | 107 (91.5%) | 111 (94.9%) | 116 (99.1%) |  |
| 1 | 21 (17.9%) ^a^ | 16 (13.7%) ^b^ | 12 (10.3%) ^b^ | 9 (7.7%) ^b^ | 6 (5.1%) ^b^ | 4 (3.4%) ^b^ |  |
| 2 | 15 (12.8%) ^a^ | 3 (2.6%) ^b^ | 1 (0.9%) ^b^ | 1 (0.9%) ^b^ | 0 (0.0%) ^b^ | 1 (0.9%) ^b^ |  |
| 3 | 3 (2.6%) ^a^ | 0 (0.0%) ^b^ | 0 (0.0%) ^b^ | 0 (0.0%) ^b^ | 0(0.0%) ^b^ | 0 (0.0%) ^b^ |  |
| 4 | 0 (0.0%) | 0 (0.0%) | 0 (0.0%) | 0 (0.0%) | 0(0.0%) | 0 (0.0%) |  |
| **Neutropenia** |  |  |  |  |  |  | <0.001 |
| 0 | 80 (68.4%) | 101 (86.3%) | 106 (90.6%) | 113 (96.6%) | 115 (98.3%) | 113 (96.6%) |  |
| 1 | 19 (16.2%) ^a^ | 7 (6.0%) ^b^ | 10 (8.5%) ^b^ | 2 (1.7%) ^b^ | 1 (0.9%) ^b^ | 4 (3.4%) ^b^ |  |
| 2 | 5 (4.3%) ^a^ | 4 (3.4%) ^b^ | 1 (0.9%) ^b^ | 2 (1.7%) ^b^ | 1 (0.9%) ^b^ | 0 (0.0%) ^b^ |  |
| 3 | 11 (9.4%) ^a^ | 3 (2.6%) ^b^ | 0 (0.0%) ^b^ | 0 (0.0%) ^b^ | 0 (0.0%) ^b^ | 0 (0.0%) ^b^ |  |
| 4 | 2 (1.7%) | 2 (1.7%) | 0 (0.0%) | 0 (0.0%) | 0 (0.0%) | 0 (0.0%) |  |
| **Thrombocytopenia** |  |  |  |  |  |  |  |
| 0 | 108 (92.3%) | 108 (92.3%) | 115 (98.3%) | 117 (100%) | 117 (100%) | 117 (100%) | 0.010 |
| 1 | 2 (1.7%) ^a^ | 0 (0.0%) ^b^ | 0 (0.0%) ^b^ | 0 (0.0%) ^b^ | 0 (0.0%) ^b^ | 0 (0.0%) ^b^ |  |
| 2 | 5 (4.3%) ^a^ | 0 (0.0%) ^b^ | 1 (0.9%) ^b^ | 0 (0.0%) ^b^ | 0 (0.0%) ^b^ | 0 (0.0%) ^b^ |  |
| 3 | 1 (0.9%) | 1 (0.9%) | 0 (0.0%) | 0 (0.0%) | 0 (0.0%) | 0 (0.0%) |  |
| 4 | 1 (0.9%) | 1 (0.9%) | 1 (0.9%) | 0 (0.0%) | 0 (0.0%) | 0 (0.0%) |  |
| **Mucositis** |  |  |  |  |  |  |  |
| 0 | 115 (98.3%) | 116 (99.1%) | 116 (99.1%) | 116 (99.1%) | 116 (99.1%) | 116 (99.1%) | 0.847 |
| 1 | 2 (1.7%) | 1 (0.9%) | 1 (0.9%) | 1 (0.9%) | 1 (0.9%) | 1 (0.9%) |  |
| 2 | 0 (0.0%) | 0 (0.0%) | 0 (0.0%) | 0 (0.0%) | 0 (0.0%) | 0 (0.0%) |  |
| 3 | 0 (0.0%) | 0 (0.0%) | 0 (0.0%) | 0 (0.0%) | 0 (0.0%) | 0 (0.0%) |  |
| 4 | 0 (0.0%) | 0 (0.0%) | 0 (0.0%) | 0 (0.0%) | 0 (0.0%) | 0 (0.0%) |  |
| **Thrombus** |  |  |  |  |  |  | 0.415 |
| 0 | 116 (99.1%) | 117 (100%) | 117 (100%) | 117 (100%) | 117 (100%) | 117 (100%) |  |
| 1 | 0 (0.0%) | 0 (0.0%) | 0 (0.0%) | 0 (0.0%) | 0 (0.0%) | 0 (0.0%) |  |
| 2 | 1 (0.9%) | 0 (0.0%) | 0 (0.0%) | 0 (0.0%) | 0 (0.0%) | 0 (0.0%) |  |
| 3 | 0 (0.0%) | 0 (0.0%) | 0 (0.0%) | 0 (0.0%) | 0 (0.0%) | 0 (0.0%) |  |
| 4 | 0 (0.0%) | 0 (0.0%) | 0 (0.0%) | 0 (0.0%) | 0 (0.0%) | 0 (0.0%) |  |
| **QTc prolongation** |  |  |  |  |  |  | 0.415 |
| 0 | 117 (100%) | 116 (99.1%) | 117 (100%) | 117 (100%) | 117 (100%) | 117 (100%) |  |
| 1 | 0 (0.0%) | 1 (0.9%) | 0 (0.0%) | 0 (0.0%) | 0 (0.0%) | 0 (0.0%) |  |
| 2 | 0 (0.0%) | 0 (0.0%) | 0 (0.0%) | 0 (0.0%) | 0 (0.0%) | 0 (0.0%) |  |
| 3 | 0 (0.0%) | 0 (0.0%) | 0 (0.0%) | 0 (0.0%) | 0 (0.0%) | 0 (0.0%) |  |
| 4 | 0 (0.0%) | 0 (0.0%) | 0 (0.0%) | 0 (0.0%) | 0 (0.0%) | 0 (0.0%) |  |
| **ALT/AST increased** |  |  |  |  |  |  | <0.001 |
| 0 | 96 (82.1%) | 107 (91.5%) | 109 (93.2%) | 115 (98.3%) | 113(96.6%) | 113(96.6%) |  |
| 1 | 20 (17.1%) ^a^ | 10 (8.5%) ^b^ | 8 (6.8%) ^b^ | 2 (1.7%) ^b^ | 4 (3.4%) ^b^ | 4 (3.4%) ^b^ |  |
| 2 | 0 (0.0%) | 0 (0.0%) | 0(0.0%) | 0 (0.0%) | 0 (0.0%) | 0 (0.0%) |  |
| 3 | 1 (0.9%) ^a^ | 0 (0.0%) ^b^ | 0(0.0%) ^b^ | 0 (0.0%) ^b^ | 0 (0.0%) ^b^ | 0 (0.0%) ^b^ |  |
| 4 | 0 (0.0%) | 0 (0.0%) | 0(0.0%) | 0 (0.0%) | 0 (0.0%) | 0 (0.0%) |  |
| **Hyperbilirubinemia** |  |  |  |  |  |  | 0.797 |
| 0 | 114 (97.4%) | 116 (99.1%) | 116 (99.1%) | 116 (99.1%) | 115 (98.3%) | 115 (98.3%) |  |
| 1 | 3 (2.6%) | 1 (0.9%) | 1 (0.9%) | 1 (0.9%) | 2 (1.7%) | 2 (1.7%) |  |
| 2 | 0 (0.0%) | 0 (0.0%) | 0 (0.0%) | 0 (0.0%) | 0 (0.0%) | 0 (0.0%) |  |
| 3 | 0 (0.0%) | 0 (0.0%) | 0 (0.0%) | 0 (0.0%) | 0 (0.0%) | 0 (0.0%) |  |
| 4 | 0 (0.0%) | 0 (0.0%) | 0 (0.0%) | 0 (0.0%) | 0 (0.0%) | 0 (0.0%) |  |
| **Infectious fever** |  |  |  |  |  |  | 0.294 |
| 0 | 110 (94.0%) | 115 (98.3%) | 116 (99.1%) | 114 (97.4%) | 116 (99.1%) | 115 (98.3%) |  |
| 1 | 1 (0.9%) | 0 (0.0%) | 1 (0.9%) | 1 (0.9%) | 0 (0.0%) | 0 (0.0%) |  |
| 2 | 2 (1.7%) | 0 (0.0%) | 0 (0.0%) | 0 (0.0%) | 1 (0.9%) | 0 (0.0%) |  |
| 3 | 4 (3.4%) | 2 (1.7%) | 0 (0.0%) | 2 (1.7%) | 0 (0.0%) | 2 (1.7%) |  |
| 4 | 0 (0.0%) | 0 (0.0%) | 0 (0.0%) | 0 (0.0%) | 0 (0.0%) | 0 (0.0%) |  |

LR, low-risk; CTCAE, common terminology criteria for adverse events;

CT, consolidation treatment; MT, maintenance treatment without mercaptopurine and methotrexate;

QTc, corrected QT interval; ALT/AST, alanine transaminase / aspartate aminotransferase; a vs. b, *P* < 0.05.

Table S7.

### Assessment of toxic side effects according to CTCAE 4.0 during consolidation and maintenance treatment phase in HR group.

| **Characteristics** | **CT-1** | **CT-2** | **MT-1** | **MT-1**  **+MM** | **MT-2** | **MT-2**  **+MM** | **MT-3** | **MT-3**  **+MM** | **MT-4** | **MT-4**  **+MM** | **MT-5** | **MT-5**  **+MM** | ***p* value** |
| --- | --- | --- | --- | --- | --- | --- | --- | --- | --- | --- | --- | --- | --- |
| **Anemia** |  |  |  |  |  |  |  |  |  |  |  | <0.001 | |
| 0 | 27  33.3% | 31  38.8% | 61  76.3% | 70  87.5% | 77  96.3% | 77  96.3% | 78  97.5% | 80  100% | 80  100% | 80  100% | 80  100% | 79  98.8% |  |
| 1 | 18 ^a^  22.2% | 24  30.0% | 11 ^b^  13.8% | 8 ^b^  10.0% | 3 ^b^  3.8% | 3 ^b^  3.8% | 1 ^b^  1.3% | 0 ^b^  0.0% | 0 ^b^  0.0% | 0 ^b^  0.0% | 0 ^b^  0.0% | 1 ^b^  1.3% |  |
| 2 | 34 ^a^  42.0% | 15 ^c^  18.8% | 4^b^  5.0% | 1^b^  1.3% | 0 ^b^  0.0% | 0 ^b^  0.0% | 1 ^b^  1.3% | 0 ^b^  0.0% | 0 ^b^  0.0% | 0 ^b^  0.0% | 0^b^  0.0% | 0 ^b^  0.0% |  |
| 3 | 2  2.5% | 10 ^a^  12.5% | 3  3.8% | 1 ^b^  1.3% | 0 ^b^  0.0% | 0 ^b^  0.0% | 0 ^b^  0.0% | 0 ^b^  0.0% | 0 ^b^  0.0% | 0 ^b^  0.0% | 0 ^b^  0.0% | 0 ^b^  0.0% |  |
| 4 | 0  0.0% | 0  0.0% | 1  1.3% | 0  0.0% | 0  0.0% | 0  0.0% | 0  0.0% | 0  0.0% | 0  0.0% | 0  0.0% | 0  0.0% | 0  0.0% |  |
| **Neutropenia** |  |  |  |  |  |  |  |  |  |  |  | <0.001 | |
| 0 | 26  32.1% | 34  42.5% | 60  75.0% | 61  76.3% | 70  87.5% | 72  90.0% | 76  95.0% | 75  93.8% | 74  92.5% | 74  92.5% | 77  96.3% | 78  97.5% |  |
| 1 | 4  4.9% | 3  3.8% | 4  5.0% | 5  6.3% | 6  7.5% | 4  5.0% | 1  1.3% | 2  2.5% | 4  5.0% | 4  5.0% | 1  1.3% | 1  1.3% |  |
| 2 | 2  2.5% | 2 ^a^  2.5% | 4  5.0% | 5 ^b^  6.3% | 3  3.8% | 2  2.5% | 1  1.3% | 3  3.8% | 1  1.3% | 2  2.5% | 2  2.5% | 1  1.3% |  |
| 3 | 6  7.4% | 4 ^b^  5.0% | 2 ^b^  2.5% | 7 ^a^  8.8% | 1 ^b^  1.3% | 2 ^b^  2.5% | 1 ^b^  1.3% | 0 ^b^  0.0% | 1 ^b^  1.3% | 0 ^b^  0.0% | 0 ^b^  0.0% | 0 ^b^  0.0% |  |
| 4 | 43 ^a^  53.1% | 37 ^b^  46.3% | 10 ^b^  12.5% | 2 ^b^  2.5% | 0 ^b^  0.0% | 0 ^b^  0.0% | 1 ^b^  1.3% | 0 ^b^  0.0% | 0 ^b^  0.0% | 0 ^b^  0.0% | 0 ^b^  0.0% | 0 ^b^  0.0% |  |
| **Thrombocytopenia** |  |  |  |  |  |  |  |  |  |  |  | <0.001 | |
| 0 | 42  51.9% | 44  55.0% | 72  90.0% | 78  97.5% | 79  98.8% | 80  100% | 79  98.8% | 80  100% | 80  100% | 80  100% | 80  100% | 80  100% |  |
| 1 | 5^a^  6.2% | 3  3.8% | 2^b^  2.5% | 0 ^b^  0.0% | 1 ^b^  1.3% | 0 ^b^  0.0% | 0 ^b^  0.0% | 0 ^b^  0.0% | 0 ^b^  0.0% | 0 ^b^  0.0% | 0 ^b^  0.0% | 0 ^b^  0.0% |  |
| 2 | 9 ^a^  11.1% | 5  6.3% | 1 ^b^  1.3% | 1 ^b^  1.3% | 0 ^b^  0.0% | 0 ^b^  0.0% | 0 ^b^  0.0% | 0 ^b^  0.0% | 0 ^b^  0.0% | 0 ^b^  0.0% | 0 ^b^  0.0% | 0 ^b^  0.0% |  |
| 3 | 15 ^a^  18.5% | 9  11.3% | 0  0.0% | 0  0.0% | 0 ^b^  0.0% | 0 ^b^  0.0% | 0 ^b^  0.0% | 0 ^b^  0.0% | 0 ^b^  0.0% | 0 ^b^  0.0% | 0 ^b^  0.0% | 0 ^b^  0.0% |  |
| 4 | 10 ^a^  12.3% | 19  23.8% | 5 ^b^  6.3% | 1 ^b^  1.3% | 0 ^b^  0.0% | 0 ^b^  0.0% | 1 ^b^  1.3% | 0 ^b^  0.0% | 0 ^b^  0.0% | 0 ^b^  0.0% | 0 ^b^  0.0% | 0 ^b^  0.0% |  |
| **Nausea** |  |  |  |  |  |  |  |  |  |  |  | <0.001 | |
| 0 | 69  85.2% | 70  87.5% | 78  97.5% | 79  98.8% | 80  100% | 79  98.8% | 80  100% | 79  98.8% | 80  100% | 80  100% | 80  100% | 80  100% |  |
| 1 | 9 ^a^  11.1% | 8  10.0% | 1 ^b^  1.3% | 1 ^b^  1.3% | 0 ^b^  0.0% | 1 ^b^  1.3% | 0 ^b^  0.0% | 1 ^b^  1.3% | 0 ^b^  0.0% | 0 ^b^  0.0% | 0 ^b^  0.0% | 0 ^b^  0.0% |  |
| 2 | 2  2.5% | 2  2.5% | 0  0.0% | 0  0.0% | 0  0.0% | 0  0.0% | 0  0.0% | 0  0.0% | 0  0.0% | 0  0.0% | 0  0.0% | 0  0.0% |  |
| 3 | 1  1.2% | 0  0.0% | 1  1.3% | 0  0.0% | 0  0.0% | 0  0.0% | 0  0.0% | 0  0.0% | 0  0.0% | 0  0.0% | 0  0.0% | 0  0.0% |  |
| 4 | 0  0.0% | 0  0.0% | 0  0.0% | 0  0.0% | 0  0.0% | 0  0.0% | 0  0.0% | 0  0.0% | 0  0.0% | 0  0.0% | 0  0.0% | 0  0.0% |  |
| **Vomiting** |  |  |  |  |  |  |  |  |  |  |  | <0.001 | |
| 0 | 69  85.2% | 69  86.3% | 78  97.5% | 80  100% | 80  100% | 80  100% | 80  100% | 80  100% | 80  100% | 80  100% | 80  100% | 80  100% |  |
| 1 | 9 ^a^  11.1% | 9  11.3% | 2 ^b^  2.5% | 0 ^b^  0.0% | 0 ^b^  0.0% | 0 ^b^  0.0% | 0 ^b^  0.0% | 0 ^b^  0.0% | 0 ^b^  0.0% | 0 ^b^  0.0% | 0 ^b^  0.0% | 0 ^b^  0.0% |  |
| 2 | 3  3.7% | 2  2.5% | 0  0.0% | 0  0.0% | 0  0.0% | 0  0.0% | 0  0.0% | 0  0.0% | 0  0.0% | 0  0.0% | 0  0.0% | 0  0.0% |  |
| 3 | 0  0.0% | 0  0.0% | 0  0.0% | 0  0.0% | 0  0.0% | 0  0.0% | 0  0.0% | 0  0.0% | 0  0.0% | 0  0.0% | 0  0.0% | 0  0.0% |  |
| 4 | 0  0.0% | 0  0.0% | 0  0.0% | 0  0.0% | 0  0.0% | 0  0.0% | 0  0.0% | 0  0.0% | 0  0.0% | 0  0.0% | 0  0.0% | 0  0.0% |  |
| 5 | 0  0.0% | 0  0.0% | 0  0.0% | 0  0.0% | 0  0.0% | 0  0.0% | 0  0.0% | 0  0.0% | 0  0.0% | 0  0.0% | 0  0.0% | 0  0.0% |  |
| **Diarrhea** |  |  |  |  |  |  |  |  |  |  |  | 0.579 | |
| 0 | 81  100% | 80  100% | 79  98.8% | 80  100% | 80  100% | 80  100% | 80  100% | 80  100% | 80  100% | 80  100% | 80  100% | 80  100% |  |
| 1 | 0  0.0% | 0  0.0% | 1  1.3% | 0  0.0% | 0  0.0% | 0  0.0% | 0  0.0% | 0  0.0% | 0  0.0% | 0  0.0% | 0  0.0% | 0  0.0% |  |
| 2 | 0  0.0% | 0  0.0% | 0  0.0% | 0  0.0% | 0  0.0% | 0  0.0% | 0  0.0% | 0  0.0% | 0  0.0% | 0  0.0% | 0  0.0% | 0  0.0% |  |
| 3 | 0  0.0% | 0  0.0% | 0  0.0% | 0  0.0% | 0  0.0% | 0  0.0% | 0  0.0% | 0  0.0% | 0  0.0% | 0  0.0% | 0  0.0% | 0  0.0% |  |
| 4 | 0  0.0% | 0  0.0% | 0  0.0% | 0  0.0% | 0  0.0% | 0  0.0% | 0  0.0% | 0  0.0% | 0  0.0% | 0  0.0% | 0  0.0% | 0  0.0% |  |
| **Mucositis** |  |  |  |  |  |  |  |  |  |  |  | 0.465 | |
| 0 | 78  96.3% | 76  95.0% | 78  97.5% | 79  98.8% | 79  98.8% | 80  100% | 79  98.8% | 80  100% | 79  98.8% | 80  100% | 80  100% | 80  100% |  |
| 1 | 1  1.2% | 2  2.5% | 1  1.3% | 1  1.3% | 0  0.0% | 0  0.0% | 1  1.3% | 0  0.0% | 1  1.3% | 0  0.0% | 0  0.0% | 0  0.0% |  |
| 2 | 1  1.2% | 2  2.5% | 1  1.3% | 0  0.0% | 1  1.3% | 0  0.0% | 0  0.0% | 0  0.0% | 0  0.0% | 0  0.0% | 0  0.0% | 0  0.0% |  |
| 3 | 1  1.2% | 0  0.0% | 0  0.0% | 0  0.0% | 0  0.0% | 0  0.0% | 0  0.0% | 0  0.0% | 0  0.0% | 0  0.0% | 0  0.0% | 0  0.0% |  |
| 4 | 0  0.0% | 0  0.0% | 0  0.0% | 0  0.0% | 0  0.0% | 0  0.0% | 0  0.0% | 0  0.0% | 0  0.0% | 0  0.0% | 0  0.0% | 0  0.0% |  |
| **Bleeding** |  |  |  |  |  |  |  |  |  |  |  | 0.576 | |
| 0 | 79  97.5% | 78  97.5% | 79  98.8% | 79  98.8% | 79  98.8% | 80  100% | 79  98.8% | 80  100% | 79  98.8% | 80  100% | 80  100% | 80  100% |  |
| 1 | 1  1.2% | 0  0.0% | 0  0.0% | 0  0.0% | 0  0.0% | 0  0.0% | 0  0.0% | 0  0.0% | 0  0.0% | 0  0.0% | 0  0.0% | 0  0.0% |  |
| 2 | 1  1.2% | 2  2.5% | 1  1.3% | 1  1.3% | 1  1.3% | 0  0.0% | 1  1.3% | 0  0.0% | 1  1.3% | 0  0.0% | 0  0.0% | 0  0.0% |  |
| 3 | 0  0.0% | 0  0.0% | 0  0.0% | 0  0.0% | 0  0.0% | 0  0.0% | 0  0.0% | 0  0.0% | 0  0.0% | 0  0.0% | 0  0.0% | 0  0.0% |  |
| 4 | 0  0.0% | 0  0.0% | 0  0.0% | 0  0.0% | 0  0.0% | 0  0.0% | 0  0.0% | 0  0.0% | 0  0.0% | 0  0.0% | 0  0.0% | 0  0.0% |  |
| **Thrombus** |  |  |  |  |  |  |  |  |  |  |  | 0.442 | |
| 0 | 80  98.8% | 80  100% | 80  100% | 80  100% | 80  100% | 80  100% | 80  100% | 80  100% | 80  100% | 80  100% | 80  100% | 80  100% |  |
| 1 | 0  0.0% | 0  0.0% | 0  0.0% | 0  0.0% | 0  0.0% | 0  0.0% | 0  0.0% | 0  0.0% | 0  0.0% | 0  0.0% | 0  0.0% | 0  0.0% |  |
| 2 | 1  1.2% | 0  0.0% | 0  0.0% | 0  0.0% | 0  0.0% | 0  0.0% | 0  0.0% | 0  0.0% | 0  0.0% | 0  0.0% | 0  0.0% | 0  0.0% |  |
| 3 | 0  0.0% | 0  0.0% | 0  0.0% | 0  0.0% | 0  0.0% | 0  0.0% | 0  0.0% | 0  0.0% | 0  0.0% | 0  0.0% | 0  0.0% | 0  0.0% |  |
| 4 | 0  0.0% | 0  0.0% | 0  0.0% | 0  0.0% | 0  0.0% | 0  0.0% | 0  0.0% | 0  0.0% | 0  0.0% | 0  0.0% | 0  0.0% | 0  0.0% |  |
| **QTc prolongation** |  |  |  |  |  |  |  |  |  |  |  | 0.006 | |
| 0 | 78  96.3% | 78  97.5% | 80  100% | 80  100% | 80  100% | 80  100% | 80  100% | 80  100% | 80  100% | 80  100% | 80  100% | 80  100% |  |
| 1 | 3^a^  3.7% | 2  2.5% | 0^b^  0.0% | 0 ^b^  0.0% | 0 ^b^  0.0% | 0 ^b^  0.0% | 0 ^b^  0.0% | 0 ^b^  0.0% | 0 ^b^  0.0% | 0 ^b^  0.0% | 0 ^b^  0.0% | 0 ^b^  0.0% |  |
| 2 | 0  0.0% | 0  0.0% | 0  0.0% | 0  0.0% | 0  0.0% | 0  0.0% | 0  0.0% | 0  0.0% | 0  0.0% | 0  0.0% | 0  0.0% | 0  0.0% |  |
| 3 | 0  0.0% | 0  0.0% | 0  0.0% | 0  0.0% | 0  0.0% | 0  0.0% | 0  0.0% | 0  0.0% | 0  0.0% | 0  0.0% | 0  0.0% | 0  0.0% |  |
| 4 | 0  0.0% | 0  0.0% | 0  0.0% | 0  0.0% | 0  0.0% | 0  0.0% | 0  0.0% | 0  0.0% | 0  0.0% | 0  0.0% | 0  0.0% | 0  0.0% |  |
| **ALT/AST increased** |  |  |  |  |  |  |  |  |  |  |  | <0.001 | |
| 0 | 59  72.8% | 69  86.3% | 65  81.3% | 63  78.8% | 67  83.8% | 70  87.5% | 75  93.8% | 77  96.3% | 80  100% | 76  95.0% | 80  100% | 79  98.8% |  |
| 1 | 20^a^  24.7% | 10^b^  12.5% | 9 ^b^  11.3% | 12  15.0% | 9 ^b^  11.3% | 8 ^b^  10.0% | 2 ^b^  2.5% | 2 ^b^  2.5% | 0 ^b^  0.0% | 1 ^b^  1.3% | 0 ^b^  0.0% | 1 ^b^  1.3% |  |
| 2 | 2  2.5% | 1  1.3% | 3  3.8% | 2  2.5% | 3  3.8% | 0  0.0% | 3  3.8% | 1  1.3% | 0  0.0% | 3  3.8% | 0  0.0% | 0  0.0% |  |
| 3 | 0  0.0% | 0^b^  0.0% | 2  2.5% | 3^a^  3.8% | 1 ^b^  1.3% | 2 ^b^  2.5% | 0 ^b^  0.0% | 0 ^b^  0.0% | 0 ^b^  0.0% | 0  0.0% | 0 ^b^  0.0% | 0 ^b^  0.0% |  |
| 4 | 0  0.0% | 0 ^b^  0.0% | 1^a^  1.3% | 0  0.0% | 0 ^b^  0.0% | 0 ^b^  0.0% | 0 ^b^  0.0% | 0 ^b^  0.0% | 0 ^b^  0.0% | 0  0.0% | 0 ^b^  0.0% | 0 ^b^  0.0% |  |
| **Hyperbilirubinemia** |  |  |  |  |  |  |  |  |  |  |  | 0.519 | |
| 0 | 79  97.5% | 80  100% | 79  98.8% | 79  98.8% | 79  98.8% | 77  96.3% | 79  98.8% | 78  97.5% | 80  100% | 79  98.8% | 80  100% | 80  100% |  |
| 1 | 2  2.5% | 0  0.0% | 1  1.3% | 1  1.3% | 1  1.3% | 3  3.8% | 1  1.3% | 2  2.5% | 0  0.0% | 1  1.3% | 0  0.0% | 0  0.0% |  |
| 2 | 0  0.0% | 0  0.0% | 0  0.0% | 0  0.0% | 0  0.0% | 0  0.0% | 0  0.0% | 0  0.0% | 0  0.0% | 0  0.0% | 0  0.0% | 0  0.0% |  |
| 3 | 0  0.0% | 0  0.0% | 0  0.0% | 0  0.0% | 0  0.0% | 0  0.0% | 0  0.0% | 0  0.0% | 0  0.0% | 0  0.0% | 0  0.0% | 0  0.0% |  |
| 4 | 0  0.0% | 0  0.0% | 0  0.0% | 0  0.0% | 0  0.0% | 0  0.0% | 0  0.0% | 0  0.0% | 0  0.0% | 0  0.0% | 0  0.0% | 0  0.0% |  |
| **Creatinine elevation** |  |  |  |  |  |  |  |  |  |  |  | 0.442 | |
| 0 | 81  100% | 80  100% | 79  98.8% | 80  100% | 80  100% | 80  100% | 80  100% | 80  100% | 80  100% | 80  100% | 80  100% | 80  100% |  |
| 1 | 0  0.0% | 0  0.0% | 0  0.0% | 0  0.0% | 0  0.0% | 0  0.0% | 0  0.0% | 0  0.0% | 0  0.0% | 0  0.0% | 0  0.0% | 0  0.0% |  |
| 2 | 0  0.0% | 0  0.0% | 0  0.0% | 0  0.0% | 0  0.0% | 0  0.0% | 0  0.0% | 0  0.0% | 0  0.0% | 0  0.0% | 0  0.0% | 0  0.0% |  |
| 3 | 0  0.0% | 0  0.0% | 1  1.3% | 0  0.0% | 0  0.0% | 0  0.0% | 0  0.0% | 0  0.0% | 0  0.0% | 0  0.0% | 0  0.0% | 0  0.0% |  |
| 4 | 0  0.0% | 0  0.0% | 0  0.0% | 0  0.0% | 0  0.0% | 0  0.0% | 0  0.0% | 0  0.0% | 0  0.0% | 0  0.0% | 0  0.0% | 0  0.0% |  |
| **Infectious fever** |  |  |  |  |  |  |  |  |  |  |  | <0.001 | |
| 0 | 62  76.5% | 63  78.8% | 74  92.5% | 77  96.3% | 78  97.5% | 78  97.5% | 80  100% | 80  100% | 80  100% | 78  97.5% | 79  98.8% | 80  100% |  |
| 1 | 0  0.0% | 0  0.0% | 1  1.3% | 0  0.0% | 0  0.0% | 0  0.0% | 0  0.0% | 0  0.0% | 0  0.0% | 0  0.0% | 0  0.0% | 0  0.0% |  |
| 2 | 1  1.2% | 1  1.3% | 1  1.3% | 1  1.3% | 0  0.0% | 0  0.0% | 0  0.0% | 0  0.0% | 0  0.0% | 0  0.0% | 0  0.0% | 0  0.0% |  |
| 3 | 17^a^  21.0% | 16  20.0% | 3^b^  3.8% | 2 ^b^  2.5% | 2 ^b^  2.5% | 2 ^b^  2.5% | 0 ^b^  0.0% | 0 ^b^  0.0% | 0 ^b^  0.0% | 2 ^b^  2.5% | 1 ^b^  1.3% | 0 ^b^  0.0% |  |
| 4 | 0  0.0% | 0  0.0% | 1^a^  1.3% | 0^b^  0.0% | 0 ^b^  0.0% | 0 ^b^  0.0% | 0 ^b^  0.0% | 0 ^b^  0.0% | 0 ^b^  0.0% | 0 ^b^  0.0% | 0 ^b^  0.0% | 0 ^b^  0.0% |  |
| 5 | 1 ^a^  1.2% | 0  0.0% | 0  0.0% | 0 ^b^  0.0% | 0 ^b^  0.0% | 0 ^b^  0.0% | 0 ^b^  0.0% | 0 ^b^  0.0% | 0 ^b^  0.0% | 0 ^b^  0.0% | 0 ^b^  0.0% | 0 ^b^  0.0% |  |

HR, high-risk; CTCAE, common terminology criteria for adverse events; CT, consolidation treatment; MT, maintenance treatment without mercaptopurine and methotrexate; MT-MM, maintenance treatment with mercaptopurine and methotrexate; QTc, corrected QT interval; ALT/AST, alanine transaminase / aspartate aminotransferase; a vs. b, *P*<0.05.

Table S8.

### Infection during induction therapy.

| **risk** | **No infection** | **Infection** | ***p* value** |
| --- | --- | --- | --- |
| **LR** | 31 (64.6%) | 86 (57.0%) | 0.350 |
| **HR** | 17 (35.4%) | 65 (43.0%) |  |
| **Total** | 48 | 151 |  |

HR, high-risk; LR, low-risk.

Table S9.

### Assessment of the correlation between risk and gene mutations in APL patients.

| **Characteristics** | **LR** | **HR** | ***p* value** |
| --- | --- | --- | --- |
| **n** | 75 | 63 |  |
| **FLT3-ITD, n (%)** |  |  | 0.001 |
| no | 65 (86.7%) | 40 (63.5%) |  |
| yes | 10 (13.3%) | 23 (36.5%) |  |
| **FLT3-TKD, n (%)** |  |  | 0.004 |
| no | 67 (89.3%) | 44 (69.8%) |  |
| yes | 8 (10.7%) | 19 (30.2%) |  |
| **NRAS, n (%)** |  |  | 0.950 |
| yes | 11 (14.7%) | 9 (14.3%) |  |
| no | 64 (85.3%) | 54 (85.7%) |  |
| **KRAS, n (%)** |  |  | 0.133 |
| no | 66 (88%) | 60 (95.2%) |  |
| yes | 9 (12%) | 3 (4.8%) |  |
| **WT1, n (%)** |  |  | 0.486 |
| no | 65 (86.7%) | 57 (90.5%) |  |
| yes | 10 (13.3%) | 6 (9.5%) |  |
| **FLT3-ITD / FLT3-TKD, n (%)** |  |  | 0.879 |
| no | 74 (98.7%) | 61 (96.8%) |  |
| yes | 1 (1.3%) | 2 (3.2%) |  |
| **FLT3-ITD / NRAS, n (%)** |  |  | 0.879 |
| no | 74 (98.7%) | 61 (96.8%) |  |
| yes | 1 (1.3%) | 2 (3.2%) |  |
| **FLT3-TKD / NRAS, n (%)** |  |  | 0.535 |
| no | 72 (96%) | 58 (92.1%) |  |
| yes | 3 (4%) | 5 (7.9%) |  |
| **KRAS / NRAS, n (%)** |  |  | 0.308 |
| no | 72 (96%) | 63 (100%) |  |
| yes | 3 (4%) | 0 (0%) |  |
| **KRAS / WT1, n (%)** |  |  | 0.740 |
| no | 72 (96%) | 62 (98.4%) |  |
| yes | 3 (4%) | 1 (1.6%) |  |

HR, high-risk; LR, low-risk;

FLT3-TKD, FMS-like tyrosine kinase 3 - tyrosine kinase domain;

FLT3-ITD, FMS-like tyrosine kinase 3 - internal tandem duplication;

NRAS, Neuroblastoma RAS viral oncogene homolog;

KRAS, Kirsten rat sarcoma viral oncogene homolog;

WT1, Wilms tumor 1.

Table S10.

### Assessment of the correlation between DS and gene mutations in APL patients.

| **Characteristics** | **DS_no** | **DS_yes** | ***p* value** |
| --- | --- | --- | --- |
| **n** | 81 | 57 |  |
| **FLT3-ITD, n (%)** |  |  | 0.286 |
| no | 59 (72.8%) | 46 (80.7%) |  |
| yes | 22 (27.2%) | 11 (19.3%) |  |
| **FLT3-TKD, n (%)** |  |  | 0.712 |
| no | 66 (81.5%) | 45 (78.9%) |  |
| yes | 15 (18.5%) | 12 (21.1%) |  |
| **NRAS, n (%)** |  |  | 0.393 |
| yes | 10 (12.3%) | 10 (17.5%) |  |
| no | 71 (87.7%) | 47 (82.5%) |  |
| **KRAS, n (%)** |  |  | 0.119 |
| no | 77 (95.1%) | 49 (86%) |  |
| yes | 4 (4.9%) | 8 (14%) |  |
| **WT1, n (%)** |  |  | 0.452 |
| no | 73 (90.1%) | 49 (86%) |  |
| yes | 8 (9.9%) | 8 (14%) |  |
| **FLT3-ITD / FLT3-TKD, n (%)** |  |  | 0.381 |
| no | 78 (96.3%) | 57 (100%) |  |
| yes | 3 (3.7%) | 0 (0%) |  |
| **FLT3-ITD / NRAS, n (%)** |  |  | 0.757 |
| no | 80 (98.8%) | 55 (96.5%) |  |
| yes | 1 (1.2%) | 2 (3.5%) |  |
| **FLT3-TKD / NRAS, n (%)** |  |  | 1.000 |
| no | 76 (93.8%) | 54 (94.7%) |  |
| yes | 5 (6.2%) | 3 (5.3%) |  |
| **KRAS / NRAS, n (%)** |  |  | 0.135 |
| no | 81 (100%) | 54 (94.7%) |  |
| yes | 0 (0%) | 3 (5.3%) |  |
| **KRAS / WT1, n (%)** |  |  | 0.057 |
| no | 81 (100%) | 53 (93%) |  |
| yes | 0 (0%) | 4 (7%) |  |

DS, differentiation syndrome;

FLT3-TKD, FMS-like tyrosine kinase 3 - tyrosine kinase domain;

FLT3-ITD, FMS-like tyrosine kinase 3 - internal tandem duplication;

NRAS, Neuroblastoma RAS viral oncogene homolog;

KRAS, Kirsten rat sarcoma viral oncogene homolog;

WT1, Wilms tumor 1.

Table S11.

### Incidence of DS in patients with or without cytarabine treatment.

| **Cytarabine** | **DS_no** | **DS_yes** | ***p* value** |
| --- | --- | --- | --- |
| **no** | 55 (45.1%) | 14 (18.2%) | <0.001 |
| **yes** | 67 (54.9%) | 63 (81.8%) |  |
| **Total** | 122 | 77 |  |

DS, differentiation syndrome.

Table S12.

### Mutation data in children with APL for specific genes.

| **ID** | **FLT3-ITD** | **FLT3-TKD** | **NRAS** | **KRAS** | **WT1** | **FLT3-ITD/TKD** | **FLT3-ITD/NRAS** | **KRAS/NRAS** |
| --- | --- | --- | --- | --- | --- | --- | --- | --- |
| **N02041511** | no | no | yes | no | no | no | no | no |
| **N02125449** | no | no | no | no | no | no | no | no |
| **N02182563** | no | no | no | no | no | no | no | no |
| **N02248248** | no | no | no | no | yes | no | no | no |
| **N10041809** | yes | no | no | no | no | no | no | no |
| **N10069587** | no | no | no | no | no | no | no | no |
| **N10082337** | no | no | no | no | no | no | no | no |
| **N10082837** | no | no | no | no | yes | no | no | no |
| **N10097223** | yes | no | no | no | no | no | no | no |
| **N10124927** | no | no | no | no | yes | no | no | no |
| **N1291698** | no | yes | no | no | no | no | no | no |
| **N1301339** | no | no | no | no | no | no | no | no |
| **N1330756** | no | no | no | no | no | no | no | no |
| **N1338246** | no | no | yes | no | no | no | no | no |
| **N1340006** | no | no | no | no | no | no | no | no |
| **N1341607** | no | no | no | no | no | no | no | no |
| **N1342592** | no | no | no | no | no | no | no | no |
| **N1344840** | no | no | no | no | no | no | no | no |
| **N1345231** | no | no | no | no | no | no | no | no |
| **N1346275** | yes | no | no | no | no | no | no | no |
| **N1347482** | yes | no | no | no | no | no | no | no |
| **N1349640** | no | yes | no | yes | no | no | no | no |
| **N1372655** | yes | no | no | no | no | no | no | no |
| **N1378518** | no | no | no | no | yes | no | no | no |
| **N1388882** | no | no | no | no | no | no | no | no |
| **N1392959** | yes | no | no | no | no | no | no | no |
| **N1396483** | no | no | no | no | no | no | no | no |
| **N1396735** | no | no | no | no | no | no | no | no |
| **N1608693** | no | no | no | no | no | no | no | no |
| **N1614771** | no | no | no | no | no | no | no | no |
| **N1756195** | yes | no | no | no | no | no | no | no |
| **N18162789** | no | no | no | no | no | no | no | no |
| **N19072607** | no | no | no | no | no | no | no | no |
| **N19076111** | no | no | no | no | no | no | no | no |
| **N19117074** | no | yes | no | no | no | no | no | no |
| **N19136747** | no | yes | no | no | no | no | no | no |
| **N19173582** | no | yes | no | no | no | no | no | no |
| **N20011958** | no | no | yes | yes | no | no | no | yes |
| **N20053464** | no | no | no | no | no | no | no | no |
| **N20066266** | yes | no | no | no | no | no | no | no |
| **N20111976** | no | yes | yes | no | no | no | no | no |
| **N20133106** | no | no | yes | no | no | no | no | no |
| **N20138562** | yes | no | no | no | no | no | no | no |
| **N20143341** | no | no | no | yes | yes | no | no | no |
| **N20149892** | no | no | no | no | no | no | no | no |
| **N20168469** | no | yes | no | no | no | no | no | no |
| **N20170203** | no | no | no | no | no | no | no | no |
| **N20175910** | no | yes | yes | no | no | no | no | no |
| **N20179200** | no | no | no | yes | no | no | no | no |
| **N20185315** | no | no | no | no | no | no | no | no |
| **N20190150** | no | no | no | no | yes | no | no | no |
| **N20192113** | yes | no | no | no | no | no | no | no |
| **N20206568** | no | yes | no | no | no | no | no | no |
| **N20218968** | no | no | no | no | no | no | no | no |
| **N20226529** | no | no | no | yes | yes | no | no | no |
| **N20249127** | yes | no | no | no | no | no | no | no |
| **N20342520** | no | yes | no | no | no | no | no | no |
| **N20350051** | no | yes | no | no | no | no | no | no |
| **N20539625** | no | no | no | no | no | no | no | no |
| **N20587869** | no | no | no | no | no | no | no | no |
| **N20682589** | no | no | no | no | no | no | no | no |
| **N20905259** | no | no | no | no | no | no | no | no |
| **N21035655** | no | yes | yes | no | no | no | no | no |
| **N21094970** | no | no | no | no | yes | no | no | no |
| **N21135818** | no | yes | yes | no | no | no | no | no |
| **N21216595** | no | no | yes | no | no | no | no | no |
| **N21299940** | no | no | no | no | no | no | no | no |
| **N21435233** | no | no | no | no | no | no | no | no |
| **N22063731** | no | no | no | no | no | no | no | no |
| **N3634659** | no | no | no | no | no | no | no | no |
| **N3927174** | no | no | no | no | no | no | no | no |
| **N41264986** | yes | yes | no | no | no | yes | no | no |
| **N533419** | no | no | no | no | no | no | no | no |
| **N536195** | yes | no | yes | no | no | no | yes | no |
| **N542224** | no | no | no | yes | no | no | no | no |
| **N543612** | no | no | no | no | no | no | no | no |
| **N561186** | yes | no | no | no | no | no | no | no |
| **N566836** | no | no | no | no | no | no | no | no |
| **N571253** | no | no | no | no | no | no | no | no |
| **N575192** | no | yes | no | no | no | no | no | no |
| **N580868** | no | no | no | no | no | no | no | no |
| **N582713** | no | yes | yes | no | no | no | no | no |
| **N587117** | yes | no | no | no | no | no | no | no |
| **N590892** | yes | no | no | no | no | no | no | no |
| **N590999** | no | no | no | no | no | no | no | no |
| **N596142** | no | no | no | no | no | no | no | no |
| **N598526** | yes | no | no | no | yes | no | no | no |
| **N628583** | yes | no | no | no | no | no | no | no |
| **N629967** | no | no | yes | no | no | no | no | no |
| **N629990** | no | yes | no | no | yes | no | no | no |
| **N640049** | no | yes | no | no | no | no | no | no |
| **N64850562** | no | no | no | no | no | no | no | no |
| **N64880146** | no | no | no | no | no | no | no | no |
| **N65057752** | yes | no | yes | no | no | no | yes | no |
| **N651460** | no | yes | yes | no | yes | no | no | no |
| **N651954** | no | no | yes | no | yes | no | no | no |
| **N664201** | no | no | no | no | no | no | no | no |
| **N671969** | no | no | no | no | no | no | no | no |
| **N677486** | no | no | no | yes | no | no | no | no |
| **N677964** | yes | no | no | no | no | no | no | no |
| **N699509** | no | no | no | yes | no | no | no | no |
| **N704925** | no | no | yes | no | no | no | no | no |
| **N705213** | yes | yes | no | no | no | yes | no | no |
| **N705998** | yes | no | no | no | no | no | no | no |
| **N713823** | yes | no | no | no | no | no | no | no |
| **N715554** | no | no | no | no | no | no | no | no |
| **N720930** | no | no | no | yes | yes | no | no | no |
| **N721625** | no | no | no | no | no | no | no | no |
| **N750779** | yes | no | no | no | yes | no | no | no |
| **N752952** | no | yes | no | no | no | no | no | no |
| **N774050** | no | no | no | no | no | no | no | no |
| **N778450** | no | no | no | no | no | no | no | no |
| **N788337** | no | no | no | no | yes | no | no | no |
| **N795180** | no | yes | no | no | no | no | no | no |
| **N800618** | no | no | no | no | no | no | no | no |
| **N802565** | no | yes | yes | yes | no | no | no | yes |
| **N807787** | no | yes | yes | no | no | no | no | no |
| **N816758** | yes | yes | no | no | no | yes | no | no |
| **N817551** | yes | no | yes | no | no | no | yes | no |
| **N8659203** | no | no | no | no | no | no | no | no |
| **N9079668** | no | no | no | no | no | no | no | no |
| **N9201518** | no | no | no | no | no | no | no | no |
| **N9213207** | yes | no | no | no | no | no | no | no |
| **N9261923** | no | yes | no | no | no | no | no | no |
| **N9317367** | no | yes | no | no | no | no | no | no |
| **N9362099** | yes | no | no | no | no | no | no | no |
| **N9414944** | no | no | no | no | no | no | no | no |
| **N9442369** | yes | no | no | yes | yes | no | no | no |
| **N9515276** | no | no | no | no | no | no | no | no |
| **N9526449** | no | no | no | no | no | no | no | no |
| **N9556943** | no | no | no | no | no | no | no | no |
| **N9678714** | no | no | no | no | no | no | no | no |
| **N9705115** | yes | no | no | no | no | no | no | no |
| **N9820020** | no | no | no | no | no | no | no | no |
| **N9925010** | no | no | yes | yes | no | no | no | yes |
| **N9940286** | yes | no | no | no | no | no | no | no |
| **N9965354** | yes | no | no | no | no | no | no | no |
| **N9980929** | yes | no | no | no | no | no | no | no |

ID, identification; FLT3-TKD, FMS-like tyrosine kinase 3 - tyrosine kinase domain; FLT3-ITD, FMS-like tyrosine kinase 3 - internal tandem duplication; NRAS, Neuroblastoma RAS viral oncogene homolog; KRAS, Kirsten rat sarcoma viral oncogene homolog;

WT1, Wilms tumor 1.
